# Liver Fibrosis—From Mechanisms of Injury to Modulation of Disease

**DOI:** 10.3389/fmed.2021.814496

**Published:** 2022-01-11

**Authors:** Christian Liedtke, Yulia A. Nevzorova, Tom Luedde, Henning Zimmermann, Daniela Kroy, Pavel Strnad, Marie-Luise Berres, Jürgen Bernhagen, Frank Tacke, Jacob Nattermann, Ulrich Spengler, Tilman Sauerbruch, Alexander Wree, Zeinab Abdullah, René H. Tolba, Jonel Trebicka, Twan Lammers, Christian Trautwein, Ralf Weiskirchen

**Affiliations:** ^1^Department of Internal Medicine III, University Hospital RWTH Aachen, Aachen, Germany; ^2^Department of Immunology, Ophthalmology and Otolaryngology, School of Medicine, Complutense University Madrid, Madrid, Spain; ^3^Medical Faculty, Department of Gastroenterology, Hepatology and Infectious Diseases, University Hospital Duesseldorf, Heinrich Heine University, Duesseldorf, Germany; ^4^Chair of Vascular Biology, Institute for Stroke and Dementia Research (ISD), Klinikum der Universität München (KUM), Ludwig-Maximilians-University (LMU), Munich, Germany; ^5^Department of Hepatology and Gastroenterology, Charité Universitätsmedizin Berlin, Campus Virchow-Klinikum and Campus Charité Mitte, Berlin, Germany; ^6^Department of Internal Medicine I, University Hospital Bonn, Bonn, Germany; ^7^Institute for Molecular Medicine and Experimental Immunology, University Hospital of Bonn, Bonn, Germany; ^8^Institute for Laboratory Animal Science and Experimental Surgery, RWTH Aachen University Hospital, Aachen, Germany; ^9^Department of Internal Medicine I, University Hospital Frankfurt, Frankfurt, Germany; ^10^Institute for Experimental Molecular Imaging, RWTH Aachen University Hospital, Aachen, Germany; ^11^Institute of Molecular Pathobiochemistry, Experimental Gene Therapy and Clinical Chemistry (IFMPEGKC), University Hospital RWTH Aachen, Aachen, Germany

**Keywords:** hepatic stellate cell, hepatocytes, inflammation, chemokines, cytokines, cirrhosis, extracellular matrix, resolution

## Abstract

The Transregional Collaborative Research Center “Organ Fibrosis: From Mechanisms of Injury to Modulation of Disease” (referred to as SFB/TRR57) was funded for 13 years (2009–2021) by the German Research Council (DFG). This consortium was hosted by the Medical Schools of the RWTH Aachen University and Bonn University in Germany. The SFB/TRR57 implemented combined basic and clinical research to achieve detailed knowledge in three selected key questions: (i) What are the relevant mechanisms and signal pathways required for initiating organ fibrosis? (ii) Which immunological mechanisms and molecules contribute to organ fibrosis? and (iii) How can organ fibrosis be modulated, e.g., by interventional strategies including imaging and pharmacological approaches? In this review we will summarize the liver-related key findings of this consortium gained within the last 12 years on these three aspects of liver fibrogenesis. We will highlight the role of cell death and cell cycle pathways as well as nutritional and iron-related mechanisms for liver fibrosis initiation. Moreover, we will define and characterize the major immune cell compartments relevant for liver fibrogenesis, and finally point to potential signaling pathways and pharmacological targets that turned out to be suitable to develop novel approaches for improved therapy and diagnosis of liver fibrosis. In summary, this review will provide a comprehensive overview about the knowledge on liver fibrogenesis and its potential therapy gained by the SFB/TRR57 consortium within the last decade. The kidney-related research results obtained by the same consortium are highlighted in an article published back-to-back in Frontiers in Medicine.

## Introduction

Fibrosis and resulting organ failure accounts for at least one third of all disease-related deaths worldwide (1). The liver and the kidney both develop excessive fibrotic tissue as a consequence of chronic diseases. Although the increasing incidence of end-stage liver and kidney disease associated with fibrosis is a major cause of morbidity and mortality and a substantial economic burden to health care systems, no specific anti-fibrotic drugs are available to date for hepatic or renal fibrosis. Consequently, there is an urgent need for a better understanding of the molecular mechanisms of fibrotic liver and kidney diseases in order to improve diagnostics and develop new treatment options. In order to foster research on liver and kidney fibrosis, the German Research Foundation (DFG) has established the Collaborative Research Center SFB/TRR57 “Organ Fibrosis: From Mechanisms of Injury to Modulation of Disease,” which was funded from 2009 to 2021. Here, we will review the liver-related proceedings made by this consortium in the context of the state-of-the-art knowledge.

The SFB/TRR57 investigated essential mechanisms of organ fibrosis in the liver. To this end, the consortium focussed on important subject areas referred to as: (i) initiation of liver fibrosis, (ii) immunological mechanisms, and (iii) repair and modulation of liver fibrosis as specified in detail further below.

## Initiation of Liver Fibrosis

**Figure 1:**
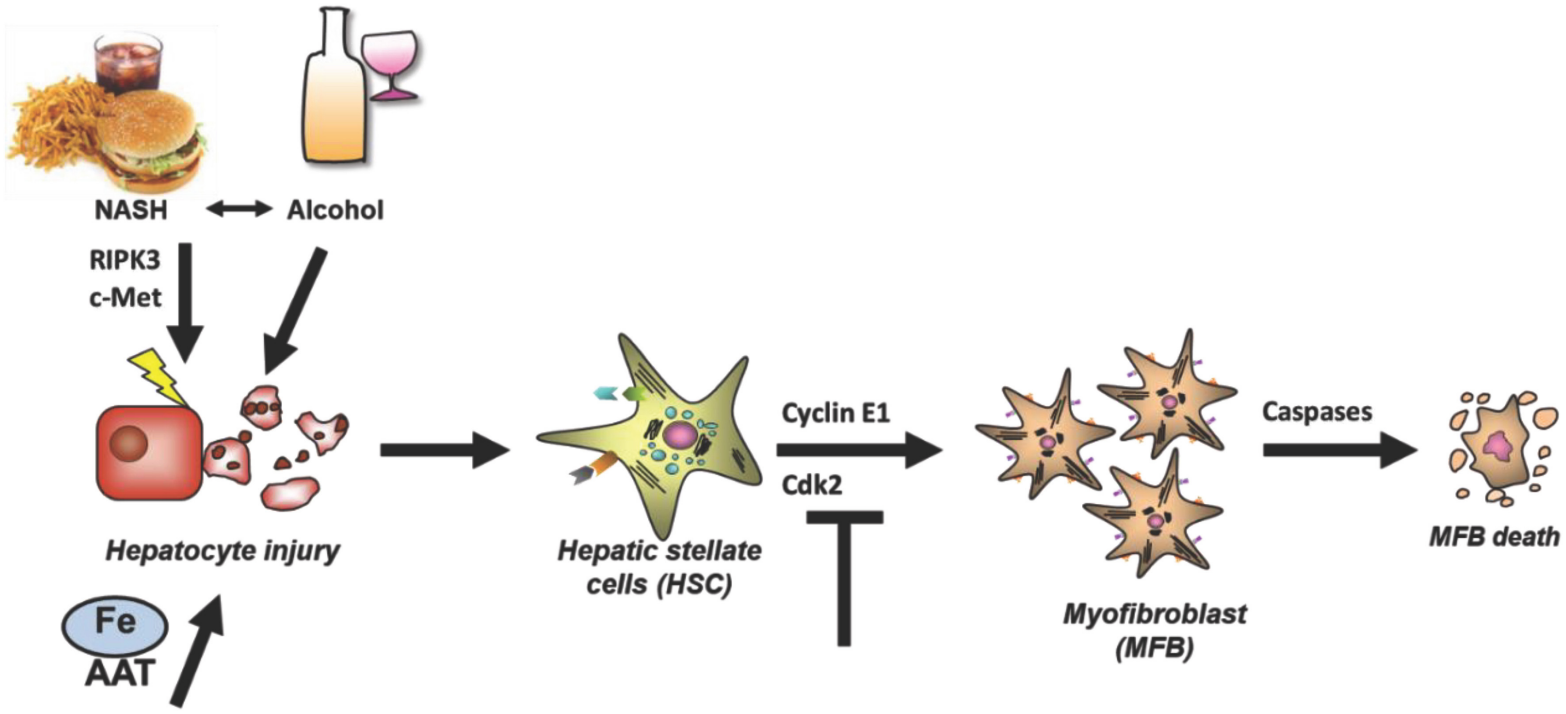
Relevant mechanisms of liver fibrosis initiation reviewed in this article. Four liver-related aspects will be reviewed which aimed to characterize and prevent fibrosis initiation at the level of hepatocyte injury or myofibroblast activation. Hepatocyte injury can be mediated by a variety of mechanisms such as alcohol, NASH, RIPK3-mediated necroptosis, impaired c-Met signaling, iron overload and AAT. In any case, death of hepatocytes triggers activation of HSCs and their transdifferentiation and proliferation of Myofibroblasts. The latter process depends on the cell cycle regulator Cyclin E1 and its interacting kinase Cdk2. It is hypothesized that fibrosis can be halted by inducing Caspase-mediated MFB cell death. AAT, α1-antitrypsin; AAT-d, α1-antitrypsin-deficiency; DAMPs, Danger-associated molecular patterns; Fe, Iron overload; NASH, non-alcoholic steatohepatitis.

Regarding initiation of liver fibrosis, in particular four mechanisms will be emphasized in this review as illustrated in Figure 1. Notable achievements included the identification of a novel risk factor for aggravation of alcoholic liver damage and liver fibrosis as well as the development of RNAi-mediated anti-fibrotic therapy concepts. Moreover, a potential role of a novel form of programmed cell death called “necroptosis” in non-alcoholic steatohepatitis (NASH) mediated fibrosis has been identified that might represent a potential drug target. We will further report on the important role of hepatocyte growth factor (HGF)/c-mesenchymal-epithelial transition receptor (c-Met)-dependent signaling during NASH fibrogenesis, and describe a novel model of iron overload-associated liver fibrosis uncovering lysosomal iron overload as a novel pro-fibrotic mechanism.

### Hepatic Cell Cycle Activity in Initiation and Progression of Liver Fibrosis

Initiation of liver fibrosis is associated with cell cycle activity of several hepatic cell entities including hepatocytes, hepatic stellate cells, endothelial cells and others. This is plausible, considering that liver fibrosis is based on chronic liver damage and liver cell death. Dying hepatocytes are replaced through compensatory hepatocyte proliferation. In addition, hepatocyte cell death and tissue injury triggers activation and proliferation of hepatic stellate cells (HSCs) as well as other hepatic cell types. Activated HSCs transdifferentiate into myofibroblasts, which continue to proliferate and produce extracellular matrix (Figure 1), which is the basis for liver scarring with fibrogenesis and the risk of disease progression toward cirrhosis as well as toward hepatocellular carcinoma (HCC). Thus, hepatic proliferation is a key event during liver fibrosis initiation and targeting cell cycle activity in the liver could be a promising anti-fibrotic treatment approach.

One key for preventing liver fibrogenesis by targeting the cell cycle machinery will be the identification of cell cycle factors that are specifically important for driving liver fibrosis without affecting general liver homeostasis or liver regeneration. Basically, the mammalian cell cycle is regulated by specific combinations of cyclin-dependent kinases (CDKs) and their regulatory subunits referred to as cyclins. Importantly, many of the known cyclins and Cdks are dispensable for overall cell cycle activity presumably due to high functional redundancy between these proteins. For instance, we have shown that single genetic loss of Cyclin E1, Cyclin E2, or Cdk2 in mice does not prevent proper liver regeneration after partial hepatectomy, and even the concomitant genetic deletion of all three genes still allows sufficient liver mass restitution to prevent mortality of hepatectomized mice (2, 3). However, this does not exclude the possibility that any of these factors might perform essential, cell type specific functions in the context of disease development. In addition to cyclins and Cdks, an abundance of other mitogens (i.e., growth factors, transcription factors) is capable of modulating and/or controlling the cell cycle activity. The proto-oncogene c-*myc* is a transcription factor that activates expression of many pro-proliferative genes including E-type cyclins and Cdk2 (4). In a recent study, it has been observed that patients with advanced liver fibrosis or liver cirrhosis showed significant up-regulation of hepatic c-*myc* gene expression (5). These findings were mirrored in experimental mouse models. Briefly, mice with over-expression of c-*myc* in hepatocytes (alb-myc^tg^) revealed increased basal liver collagen disposition and spontaneous HSC activation. Importantly, primary HSC derived from alb-myc^tg^ mice showed enhanced proliferation and accelerated transdifferentiation into myofibroblasts *in vitro*. Accordingly, fibrosis initiation *in vivo* after chronic carbon tetrachloride (CCl_4_) treatment was accelerated in alb-myc^tg^ mice compared to controls. Mechanistically, some data pointing to a paracrine crosstalk of c-*myc* over-expressing hepatocytes and HSC as livers of alb-myc^tg^ mice revealed increased levels of PDGF-B. As a consequence, over-expression of c-*myc* in hepatocytes triggered accelerated experimental liver fibrogenesis.

The sequels of elevated hepatic c-*myc* expression were also evaluated in the clinical relevant setting of liver fibrogenesis due to alcoholic liver disease (ALD). It has been demonstrated that c-*myc* was induced in human and murine (i.e., experimental) ALD (6). Moreover, patient-derived data clearly showed a significant correlation of hepatic c-*myc* expression with the strength of ALD progression. Overall, expression of c-*myc* and alcohol-uptake synergistically accelerated the progression of ALD, and additional data strongly indicated that this was most likely due to a loss of p53-dependent protection mechanisms (6). Thus, c-*myc* can be considered a new potential marker for the early detection of ALD and for the identification of risk patients. Unfortunately, due to its complex functions in almost all cellular processes, c-*myc* is still considered an undruggable target. However, several approaches are currently under development which could help to modulate c-*myc* dependent effects in the future (7).

Another aspect of cell cycle regulation during liver fibrosis initiation has become evident in the last years by focussing on the pro-fibrotic role of E-type cyclins. Of note, Cyclin E/Cdk2 kinase was shown to be regulated by c-*myc* in several independent studies (8–10). In good agreement with the published investigations on the role of c-*myc*, increased expression of Cyclin E1—but not of Cyclin E2 has been shown in human and murine liver fibrosis (11). In humans, Cyclin E1 mRNA expression was significantly up-regulated in patients with advanced hepatic fibrosis and liver cirrhosis, compared to healthy control livers or patients with mild fibrosis. In contrast, Cyclin E2 was not aberrantly expressed in liver fibrosis at any stage. In mice, Cyclin E1, but not Cyclin E2 was induced in the liver after repeated treatment with the fibrosis-inducing toxin CCl_4_, and correlated with the strength of fibrosis progression. Genetic experiments revealed that the constitutive inactivation of Cyclin E1 prevents the initiation at least of experimental liver fibrosis in mice. Further investigations showed that Cyclin E1 is essential for cell cycle progression, transdifferentiation and survival of HSCs suggesting that HSCs are a key effector cell of the pro-fibrogenic function of Cyclin E1. As Cyclin E1 is not required for liver regeneration (2), these findings suggest that its therapeutic inhibition could reduce liver fibrogenesis without affecting the regenerative capacity of the liver.

Advanced studies then addressed whether these key findings could be translated into a pre-clinical therapeutic approach. So far, pharmacological small-molecule inhibitors directly targeting Cyclin E1 have not been developed; however, there are several groups of inhibitors available targeting the kinase activity of Cdk2. Yet, it has to be mentioned that these inhibitors are not completely specific to Cdk2 but also mediate off-target effects on other Cdks such as Cdk1, Cdk5, 7, 9 and others (12). In order to treat experimental liver fibrosis by targeting Cyclin E/Cdk2, basically two different strategies have been used. For targeting Cyclin E1 *in vivo*, liposome-based delivery of Cyclin E1- specific small interfering RNA (siRNA) was applied, whereas for targeting Cdk2 in HSCs, the efficacy of the second-generation pan-Cdk inhibitor CR8 (13) was tested in a series of *in vitro* experiments. Importantly, both approaches turned out to be highly promising and will be briefly reviewed below.

In wild type (WT) mice, systemic delivery of stabilized siRNA, using a liposome-based carrier, targeted ~95% of HSCs, 70% of hepatocytes, and 40% of CD45-positive leukocytes after single injection, and the accumulation of siRNA after 24 h was mainly limited to the liver (14). Importantly, ubiquitous delivery of Cyclin E1 siRNA to all liver cells turned out to be strongly beneficial during acute toxic liver injury and in the course of hepatic fibrogenesis. Major side effects regarding toxicity or survival were not observed using this strategy. In more detail, it could be demonstrated that preventive systemic delivery of stabilized CcnE1-siRNA *in vivo* only once a week was sufficient to inhibit Cyclin E1 induction in settings of both, acute and chronic liver injury, and this was sufficient to prevent the initiation of experimental liver fibrosis. For the underlying mechanisms it has been suggested that the CcnE1-siRNA primarily prevents proliferation and survival of activated HSCs during chronic fibrosis progression thereby reducing the formation of extracellular matrix. In addition, it has been shown that CcnE1-siRNA reduces the proliferation and infiltration of pro-fibrotic leukocytes in the challenged liver and thereby may attenuate the overall inflammatory response after a pro-fibrotic stimulation.

However, in a real clinical situation, a patient would only be treated after the development of liver fibrosis. It is therefore important to note that Cyclin E1 siRNA was shown to still act anti-fibrotic, if it is administered after induction of liver fibrosis. In conclusion, this approach could basically be a promising treatment option for patients with liver fibrosis in the future (14). In this context it is very promising to note that currently two independent siRNA-based therapeutics have been approved by the U. S. Food and Drug Administration (FDA) (15, 16), which suggests that RNA-interference is a suitable technology for the treatment of diseases in humans.

The characterization of the pleiotropic Cdk inhibitor CR8 for its anti-fibrotic properties was so far restricted to *in vitro* analyses on established immortalized HSC lines, primary HSCs and primary hepatocytes (17). CR8 treatment of HSCs resulted in cell cycle arrest, apoptosis and down-regulation of pro-fibrotic genes. In contrast, it has been shown that hepatocytes tolerate substantially higher doses of CR8 than HSCs without prominent cytotoxic effects. Therefore, this proof-of-concept study demonstrated that pharmacological Cdk-inhibition restricts the pro-fibrotic properties of HSCs, while preserving the regeneration capacity of hepatocytes under the same conditions. Thus, CR8 and related drugs might be promising therapeutic agents for the treatment of liver fibrosis. However, it needs to be addressed in the future if other non-parenchymal liver cells such as Kupffer cells, infiltrating immune cells or biliary epithelial cells will also tolerate CR8 to the same extend as hepatocytes. In addition, this concept will need intensive *in vivo* validations in the future before pan-Cdk inhibitors can be considered for anti-fibrotic therapies.

**Figure 2:**
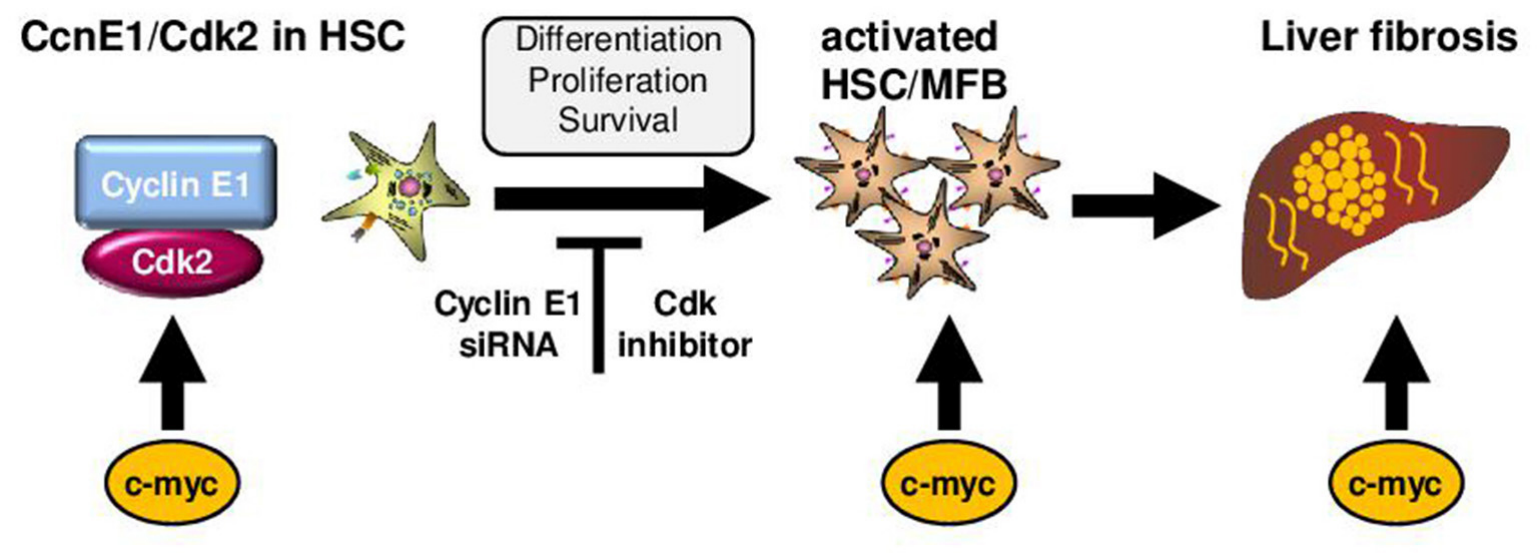
Role of cell cycle mediators for liver fibrosis initiation. Initiation of liver fibrogenesis is associated with increased hepatic cell cycle activity in HSCs. Important cell cycle mediators in these processes are c-myc and the Cyclin-dependent kinase 2 (Cdk2) in complex with its regulatory subunit Cyclin E1. In general, c-myc is involved in activation of Cyclin E1/Cdk2. Moreover, over-expression of c-myc in hepatocytes triggers activation of HSCs and is observed in patients and mice with liver fibrosis. Cyclin E1 is indispensable for initiation of liver fibrosis and also essential for proliferation, differentiation and survival of HSCs. Direct targeting of Cyclin E1 is currently feasible by using RNA interference (siRNA) *in vivo*. Anti-fibrotic effects in HSCs can also be triggered by the use of pharmacological inhibition of Cdk-activity.

The findings on the role of cell cycle activation in liver fibrogenesis are summarized in Figure 2: Initiation of liver fibrogenesis is associated with increased cell cycle activity involving the proto-oncogene c-*myc* and Cyclin E1. The latter one act in complex with its kinase subunit Cdk2. Overexpression of c-*myc* is observed in patients with liver fibrosis of several etiologies including ALD; yet it is considered undruggable at present due to its numerous functions. Cyclin E1 is indispensable for initiation of liver fibrosis and also essential for proliferation, differentiation and survival of HSCs. Direct targeting of Cyclin E1 at present is only feasible by using RNA interference, but was shown to act highly anti-fibrotic in mice without any notable side effects. It needs to be evaluated in future if this can be developed into a human siRNA therapeutic. Finally, anti-fibrotic effects in HSCs can also be triggered by the use of pharmacological inhibition of Cdk-activity without major effects on hepatocyte viability at least *in vitro*. However, before Cdk inhibitors can be considered for anti-fibrotic therapies in humans, an abundance of *in vivo trials* need to be performed. It is concluded that the modulation of the hepatic cell cycle activity could be general approach for the treatment of patients with moderate liver fibrosis.

### Programmed Cell Death in Non-alcoholic Steatohepatitis and Fibrosis

Non-alcoholic fatty liver disease (NAFLD), now the most prevalent liver disease in Western countries, represents an enormous threat to our health care systems. NASH, the chronic progressive manifestation of NAFLD, is the second leading risk factor for hepatocellular carcinoma (HCC), the most frequent form of liver cancer (18). Currently, there are no existing pharmacological treatment options for NASH, therefore the current treatment is primarily aimed at lifestyle change with more exercise and modification of food intake.

The occurrence of cell death—e.g., caused by lipotoxicity—represents a critical event in NASH. It triggers the recruitment of immune cells and consequently drives disease progression toward fibrosis and liver cancer development (19). How cell death and inflammation are associated with the malignant transformation of hepatocytes is currently poorly understood. In the past, apoptosis, for decades a synonym for programmed cell death, was considered the main driver for the development of NASH (20). However, recent work revealed that key molecules of necroptosis—the programmed form of necrosis—mediated by receptor interacting protein kinase (RIPK) 1, RIPK3 and mixed lineage kinase domain-like pseudokinase (MLKL)—are major contributors in the pathophysiology of NASH, fibrosis and liver cancer development (21–23). Necroptosis is a type of cell death whose features are similar to apoptosis and necrosis. However, it is initiated by ligand binding to the tumor necrosis factor receptor 1 (TNFR1), thereby forming a specific complex with capacity to initiate different downstream cascades in which the cells rupture and leak their content into the intercellular space (24). Necroptosis plays a vital role in different stages of liver disease including cholestatic liver disease, alcoholic liver disease, NASH, viral hepatitis, and liver cancer (19, 25).

As such, it was shown, that in livers of NASH patients, apoptosis is only negligibly activated while the necroptosis mediator RIPK3 is strongly overexpressed (26, 27). Of note, genetic inhibition of RIPK3 resulted in a very effective inhibition of liver fibrosis in a murine model of NASH [methionine/choline-deficient (MCD) diet], whereas apoptosis inhibition even increased disease progression (26, 28).

In contrast to the MCD model, *Ripk3* deficient mice fed with a high fat diet (HFD), exhibited more steatosis, fibrosis, and inflammation compared to HFD-treated WT mice (29). Moreover, *Ripk3* deficient mice showed an aggravation of systemic insulin resistance and increased compensatory adipocyte apoptosis in the choline deficient-high fat diet (CD-HFD) model (27). Interestingly, aggravated insulin resistance was reverted to WT conditions upon additional inhibition of apoptosis, probably in adipocytes (27). Thus, RIPK3 may have a protective, counterbalancing function in adipose tissue. This could explain the observed increased expression of *RIPK3* in adipose tissue of obese patients (27). Consistent with RIPK3, increased expression of RIPK1 was found in adipose tissue of obese patients (30). Thus, genetic knock out of *Ripk1* using antisense oligonucleotides in HFD treated wild type mice resulted in reduced diet-induced obesity and adipose tissue inflammation and improved insulin resistance. These protective effects were independent of a kinase activity, as RIPK1^K45A^ kinase dead knock in mice did not improve insulin resistance or reduce obesity on HFD feeding (30). However, Tao et al. observed less liver injury, less steatosis and decreased inflammation in HFD- and MCD-fed RIPK1^K45A^ mice (31). Further investigations revealed that RIPK1′ kinase activity in hematopoietic-derived macrophages contributed mostly to the disease progression in NASH (31).

Finally, the terminal executor of necroptosis, MLKL was found activated in livers of NASH patients (32). In contrast to RIPK3, recent studies showed protective effects in the previous mentioned high fat models upon genetic deletion of *Mlkl*. As such, Saeed et al. reported reduced hepatic steatosis and inflammation in *Mlkl*-deficient HFD-fed mice (33), as well as enhanced hepatic insulin sensitivity (34). A further study using the Western diet (FFC diet, high in fat, fructose and cholesterol), also showed reduced liver injury and hepatic steatosis upon genetic ablation of *Mlkl*, probably through a necroptosis-independent but autophagy-dependent mechanism (35).

**Figure 3:**
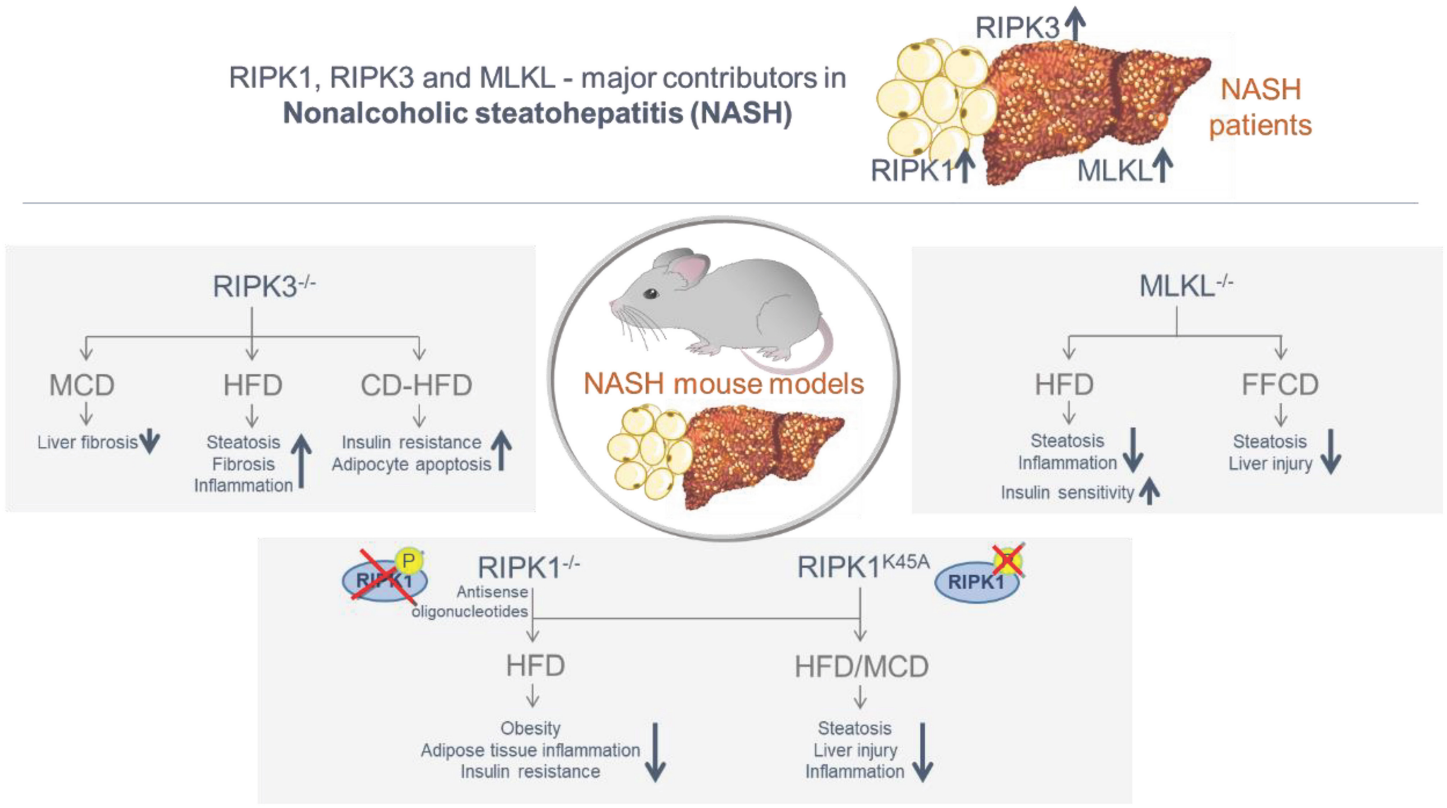
Summary of the involvement of the necroptosis-associated proteins RIPK1, RIPK3 and MLKL in obese/NASH patients and in murine NASH models. The necroptosis-associated proteins RIPK1, RIPK3, and MLKL are overexpressed in the liver and/or adipose tissue of NASH patients. Murine NASH studies with distinct knock-in and knock-out mouse models confirmed an essential function of RIPK1, RIPK3, and MLKL in the transition from NAFLD to NASH and NASH-fibrosis, including both protective and inducing mechanisms. While RIPK3 deletion led to a decrease in fibrosis in the MCD model, NASH progression was observed in the HFD and CD-HFD models. Deletion of RIPK1 and MLKL was predominantly protective in the MCD and HFD models.

Together, these studies suggest that the necroptosis-associated proteins are overexpressed in human liver and/or adipose tissue and that they are involved in the transition from NAFLD to NASH and NASH-fibrosis in mice (Figure 3). Moreover, there is evidence that the respective involvement of RIPK1, RIPK3, and MLKL may be independent of their primary function in necroptosis activation. It is so far completely unclear, whether necroptosis is executed *in vivo* at all and under which pathological circumstances. The metabolic studies carried out with the different knockout mice represent a helpful tool to approach this issue, but despite the fact that necroptosis-independent functions have been identified for all three proteins, no final conclusion can be given whether necroptosis is relevant in NASH disease. Here, an *in vivo* visualization in real time would be required, showing the execution of necroptosis with the typical associated morphological criteria. Moreover, since constitutive knockout mice were used in most murine studies, the specific function in the individual organs is still not evident. Therefore, it would be important to repeat the studies with cell type-specific conditional knockout mice in order to more precisely describe the specific function of RIPK1, RIPK3, and MLKL in NASH-fibrosis.

### Defining HGF/c-Met Dependent Diagnostic Markers and Novel Therapeutic Targets in NASH-Development

c-Met acts as the cellular receptor for hepatocyte growth factor (HGF), thereby regulating cell growth, motility and morphogenesis. This growth factor is mainly produced by cells of mesenchymal origin and it acts on epithelial and endothelial cells. Originally, c-Met has been identified as an oncogene. It serves as an important regulator for stem cell growth during embryonic development and in particular also for the growth of hepatocytes. In this regard, it is of interest that HGF knockout mice as well as mice deficient for its receptor c-Met display severe developmental defects and both die between day 13 and 16 of embryonic development. The lack of both genes results in impaired placental and liver development (36, 37). Binding of HGF to c-Met induces dimerization and phosphorylation of the receptor and lead to recruitment of intracellular adapter proteins that bind to c-Met and lead to activation of specific intracellular signaling cascades. Those activate PI3K, Ras and ERK-dependent signaling pathways and control HGF-induced pro-mitogenic and anti-apoptotic events. The latter are of particular importance, as interference with apoptosis regulating pathways can have deleterious consequences for the whole organism. Usually, the apoptosis inducing death-receptor Fas is sequestered by c-Met. This prevents an uncontrolled Fas-ligand induced death-receptor activation and apoptosis is inhibited (38). This control mechanism is disturbed during NASH pathogenesis. Fas-Ligand is produced in excess and the physiological inhibition through c-Met is hampered (39). As a consequence, apoptosis is induced and liver damage occurs (40). Thus, HGF/c-Met has direct implications for the pathogenesis of NASH and seems to be hepatoprotective. Further light into the manifold c-Met controlled activities that regulate liver physiology became evident through the introduction of conditional c-Met knockout mice by Borowiak et al. (41). Here, mice carrying the Mx-cre-induced c-Met deletion displayed a reduced liver regeneration. Analysis of hepatocellular cell cycle progression in conditional Met knockout mice indicated a defective exit from quiescence and diminished entry into S-phase. This was accompanied by a reduced activation of Erk1/2 kinase, while Akt-phosphorylation was still intact—highlighting the importance of cytokine induced signaling cross talks.

Studying the role of c-MET in murine NASH models revealed that hepatocyte specific c-Met knockout mice (c-Met^Δ*hepa*^) exhibited increased steatosis and liver infiltration upon a methionine-choline deficient (MCD) diet compared to wild type controls (c-Met^loxP/loxP^). This was accompanied by an outstanding upregulation of genes involved in fatty acid metabolism, generation of reactive oxygen species and enhanced fibrosis. Interestingly, c-Met^Δ*hepa*^ mice also display more apoptosis in the liver which could be reverted in c-Met/Caspase-8 double knockout animals, emphasizing the prominent role of c-Met in the regulation of cell survival (42). Of note, a later publication could indicate that the protective role of c-Met is not restricted to hepatocytes since genetic deletion of c-Met in Kupffer cells/macrophages, myofibroblasts and CK19^+^ cells also attenuated steatohepatitis and fibrosis in the MCD-model, highlighting a more global role of c-Met beyond liver parenchymal cells (43). Noteworthy, the mineralocorticoid receptor (MR), which has been implicated in the pathogenesis of insulin resistance and type 2 diabetes, on myeloid cells (i.e., macrophages), might contain protective HGF/c-Met signaling in hepatocytes whereby these two cells could be functionally linked in the formation of steatohepatitis and related fibrosis (44). In line with hepatoprotective consequences of c-Met signaling in experimental NASH models, Pioglitazone, a ligand of peroxisome proliferator-activated receptor gamma (PPARγ), which was shown to alleviate human steatohepatitis in clinical trials, acts through activation of c-Met (45).

Accordingly, it has also been demonstrated that the c-Met ligand HGF counteracts steatohepatitis since mice with transgenic HGF overexpression were largely protected from the deleterious effects of MCD-diet by amelioration of fibrosis and inflammation and protection from oxidative stress (46). In a subsequent study the hepatoprotective effect of HGF in NASH could be attributed to the JAK2-STAT3 pathway thereby limiting inflammation (47). Furthermore, HGF exerts anti-oxidative properties by inducing glutathione and related enzymes (48) and dampens insulin resistance and liver triglyceride and cholesterol content which might involve nuclear receptors such as the Farnesoid X receptor (FXR) (49). Blocking c-Met mitigated the beneficial effects of HGF indicating the importance of HGF/c-Met downstream signaling. Furthermore, an earlier pivotal study indicated that the HGF/c-MET axis suppresses hepatic glucose uptake promoting insulin responsiveness by direct engagement of the insulin receptor (INSR) in a INSR/c-Met complex (50). The potential role of recombinant HGF in the treatment of NASH was also demonstrated by Yang et al., who could show that mice which were fed a choline-deficient amino acid defined diet (CDAA) display improved inflammation, steatosis and lipid profile when treated with recombinant feline HGF (51).

As already mentioned, oxidative stress plays a critical role during the progression from simple steatosis toward manifest steatohepatitis. The transcription factor termed nuclear factor-E2-related factor-2 (NRF2) serves as a cellular sensor for oxidative stress. NRF2 itself is sequestered in the cytosol by Kelch-like ECH-associated protein (Keap1). During oxidative challenge, modification of Keap1 sulfhydryl groups results in the stabilization and nuclear translocation of NRF2 (52). NRF2 is crucial for antioxidant, responsive element/electrophile-responsive element (ARE/EpRE)-mediated induction of detoxifying enzymes, anti-oxidative stress genes and other target genes involved in cellular protection. Activation of these target genes serves to decrease the oxidative burden of the cells (53). This seems to be pathogenetically related to the development of NASH, as *Nrf*2^−/−^ mice are more prone to develop fatty liver degeneration (54). More refined investigations of related molecular mechanisms and potential cross talks to other signaling pathways involved in NASH pathogenesis are still scant and need to be better explored. Given this role as a master-regulator, pharmacologic or genetic inhibition of Keap1 function results in constitutive activation of *Nrf2* signaling that has been shown to exert hepato-protection against chemical-induced cytotoxicity, ischemia/reperfusion injury and alcohol-induced liver steatosis (55, 56). However, in spite of multiple published reports, results supporting an involvement of this transcription factor in the modulation of hepatic lipid accumulation and steatohepatitis remain deeply controversial. Indeed, whereas genetic *Nrf2* deletion has been demonstrated to accelerate the transition from simple steatosis to NASH (57), genetic activation of *Nrf2* resulted in different and sometimes contradictory effects. These results seem to depend on the site of Keap1 deletion, the experimental NASH model and the experimental conditions (58). Indeed, Nrf2 over-activation in hepatocyte-specific Keap1 knockout mice results in amelioration of steatosis but does not affect liver inflammation and fibrosis (59). A recent study highlighted the potential synergistic hepatoprotective role of c-Met and NRF2 in NASH by investigating the consequences of combined c-Met/Keap1 gene deletion in hepatocytes in rodent models of steatohepatitis. It was clearly demonstrated that double-knockout mice exhibit less inflammation, steatosis and oxidate stress in comparison to c-Met^Δ*hepa*^ animals indicating that the anti-oxidative factor NRF2 can override the detrimental effects of c-Met deficiency by restoring the cellular redox homeostasis (60). This finding emphasizes the crucial role of reactive oxygen species for the development and progression of steatohepatitis.

Despite these promising findings, hepato-cancerogenic effects of HGF/c-Met and Keap1/Nrf2 activity currently limit the therapeutic exploitation of the potential beneficial properties of HGF/c-Met and NRF2 in human NASH. For example, aberrant NRF2 activation has been identified to accelerate the development of hepatocellular carcinoma (HCC) from pre-cancerous lesions (61) and the HGF/c-Met axis is fundamentally involved in HCC pathogenesis *via* canonical and non-canonical pathways (62). More research is therefore warranted to unravel potential applications of c-Met and Nrf2 agonists in fatty liver disease.

### Novel Insights Into the Alpha1-Antitrypsin Deficiency-Related Liver Disease

Alpha1-antitrypsin deficiency (AATD) is a genetic condition resulting from mutations in the alpha1-antitrypsin (AAT) gene. Out of the >100 variants described to date, Pi^*^Z, resulting from a substitution of glutamate at position 342 to lysine, is the most relevant one. It results in a rapid degradation of about 70% of the synthesized protein, while 15% is secreted and another 15% forms polymers. The latter fraction gives rise to roundish globules that can be visualized in periodic acid–Schiff–diastase staining or by the appropriate immunohistochemistry. These aggregates constitute the histological hallmark of the disease (63).

Heterozygous and homozygous presence of this variant is termed as Pi^*^MZ and Pi^*^ZZ genotype, respectively, while Pi^*^MM refers to individuals without AAT mutations. Pi^*^ZZ genotype is found in around 1:3,000 individuals of European descent and is the cause of classic, severe AATD, whereas Pi^*^MZ is seen in up to 1:28 individuals in certain populations (63). Pi^*^ZZ subjects have markedly decreased serum AAT levels and are strongly predisposed to both, early onset lung emphysema as well as pediatric and adult liver disease. Recent work suggests that significant liver fibrosis develops in 20–36% of Pi^*^ZZ individuals, especially in individuals with risk factors such as male sex, older age or presence of metabolic syndrome (64, 65). Pi^*^ZZ subjects more commonly display severe liver steatosis and have decreased serum triglyceride levels, which points toward impaired lipid metabolism (65). Several non-invasive techniques have been shown as useful surrogates to identify Pi^*^ZZ subjects with significant liver involvement. Among them, liver stiffness measurement by transient elastography and determination of aspartate aminotransferase to platelet ratio index (APRI) in serum might be particularly suitable to estimate the amount of histological liver fibrosis (64–66). Regular liver ultrasounds are recommended for individuals with advanced liver fibrosis as a method for HCC surveillance (67).

People with Pi^*^MZ genotype have normal or mildly decreased serum AAT levels and are at much lower risk of developing a pathogenic liver fibrosis than Pi^*^ZZ individuals, but are still more susceptible to liver scaring than non-carriers, in particular when simultaneously suffering background liver disease (68, 69). In these conditions, Pi^*^Z carriage confers numerically higher risks for liver cirrhosis that the established genetic variants such as PNPLA3 p.I148M or TMGSF2 p.E167K (69). As a further evidence of an pathogenetic role AATD even in heterozygotes is an increased susceptibility of Pi^*^MZ individuals for development of significant liver fibrosis in case of additional metabolic stresses, obesity or presence of diabetes (69).

As a sign of AAT accumulation, PAS-D positive aggregates are seen in a vast majority of Pi^*^ZZ individuals, whereas they are found only in ~40% of Pi^*^MZ subjects (69). In line with that intrahepatic AAT levels are markedly increased in Pi^*^ZZ individuals, while they are largely unaltered in Pi^*^MZ subjects (69). However, the numbers have to be interpreted with caution since subjects with higher fibrosis stages typically display more inclusions (64, 69). Further research is needed to clarify, whether this association reflects the inability of liver-sick individuals to handle misfolded proteins or whether the protein accumulation indeed increases with progressive liver fibrosis.

**Figure 4:**
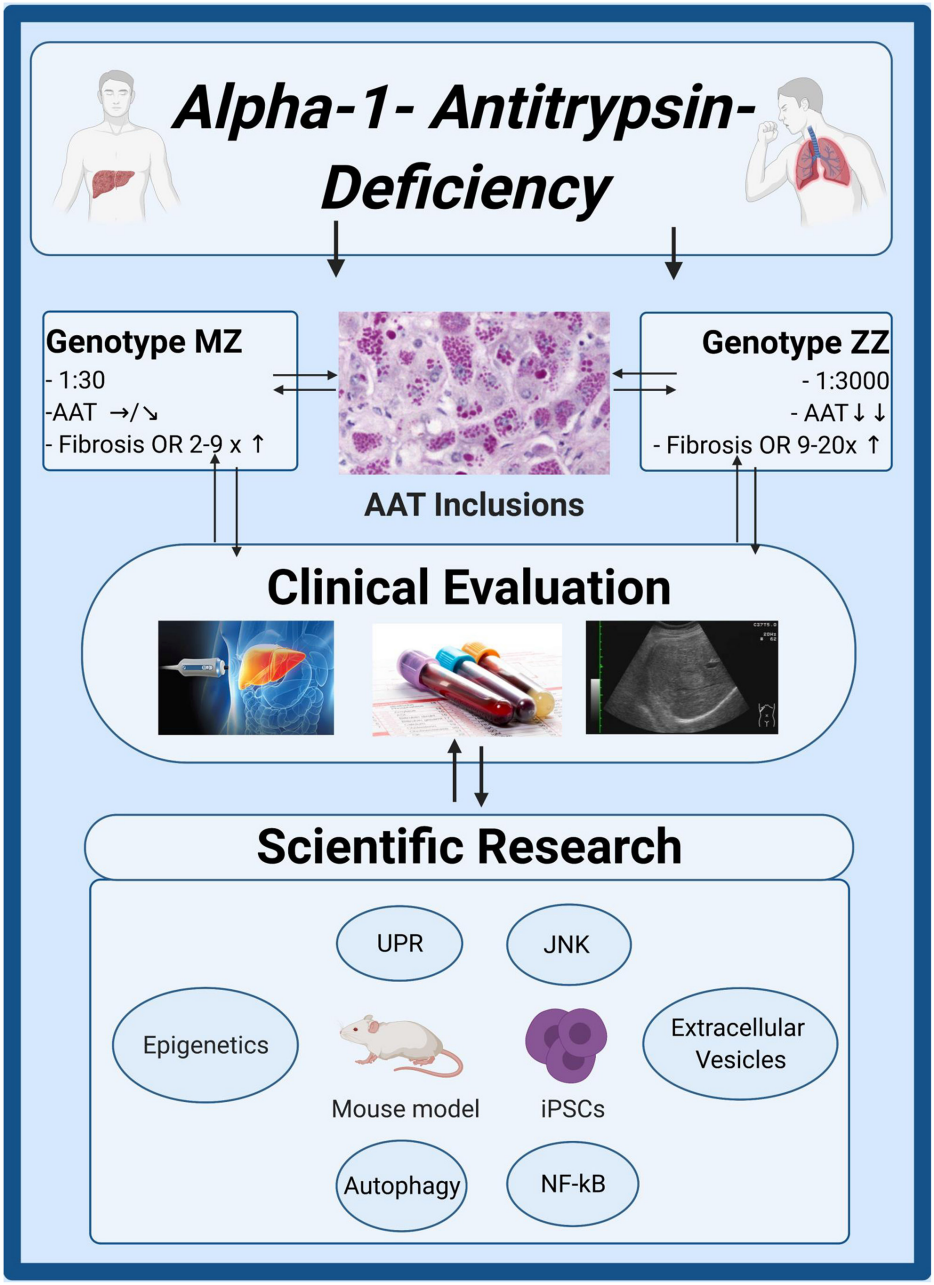
Clinical and scientific insights in alpha1-antitrypsin deficiency. Genotype MZ/ZZ refers to a presence of a heterozygous/homozygous Pi*Z mutation in alpha1-antitrypsin (AAT) gene. The upper boxes describe the frequency of both genotypes in Caucasians, the resulting serum AAT levels and their risk to develop advanced liver fibrosis compared to individuals without AAT mutations. Non-invasive techniques such as liver stiffness measurement by transient elastography or determination of aspartate aminotransferase to platelet ratio index (APRI) are suitable to estimate the amount of histological liver fibrosis. Liver ultrasounds are recommended for individuals with advanced liver fibrosis as a method of HCC surveillance. Several molecular pathways (NF-κB, JNK), protein degradation machineries (UPR, autophagy) as well as epigenetic modifications or appearance of extracellular vesicles with pro-fibrogenic cargo may contribute to disease developments. Created with BioRender.com. iPSCs, induced pluripotent stem cells; JNK, C-Jun N-terminal kinase; NF-κB, nuclear factor 'kappa-light-chain-enhancer' of activated B-cells; UPR, unfolded protein response.

While the above work defines the amount of risk associated with the carriage of Pi^*^Z variant, it also reveals a marked phenotypic heterogeneity, that makes it challenging to study the disease pathogenesis. With regard to the latter, much of our current understanding stems from mice overexpressing Pi^*^Z variant, that uncovered the importance of autophagic degradation as well as stress-associated signaling pathways such as NF-κB or JNK in the disease pathogenesis (Figure 4) (70–72). However, compared to humans, these mice express much higher PI^*^Z levels and also retains their endogenous AAT production. As a consequence, they frequently develop liver fibrosis and even liver tumors that are seen only in a small fraction of humans (70). In contrast, the human AATD-related liver disease seems to be a combination of the monogenic disorders with an additional hit, either acquired such as metabolic comorbidity or inherited. To reflect the latter, several groups started to study hepatocyte-like cells derived from patient induced pluripotent stem cells (iPSCs). These cells provided several important insights such as up-regulation of inflammation and unfolded protein response (73). They also revealed that individuals with AATD-related liver disease display a marked delay in the rate of Pi^*^Z degradation compared to liver healthy-subjects, that likely leads to appearance of globular inclusions (74).

Since animal models and iPSC cells represent only an approximation of the situation occurring in the human liver, it is imperative to complement these analyses by examination of human liver tissues, which are, however, very scarce. As an example of this effort, a systematic analysis of DNA methylation was carried out in human livers. Compared to other liver diseases, AATD livers displayed a significant genomic hypomethylation in several genes. In addition, unique epigenetic signatures that corresponded to various hallmarks of AATD have been identified (75). In plasma, AATD-individuals had a distinct population of extracellular vesicles with pathological cytokine and miRNA contents. Notably, when cultured with hepatic stellate cells, these vesicles induced an expression of fibrosis-promoting genes (76). In summary, while a substantial progress has been made in the clinical characterization of the AATD-related liver disease, the exact pathomechanisms underlying the disease development in a subset of individuals still remain to be further elucidated.

## Immunological Mechanisms in Liver Fibrosis

**Figure 5:**
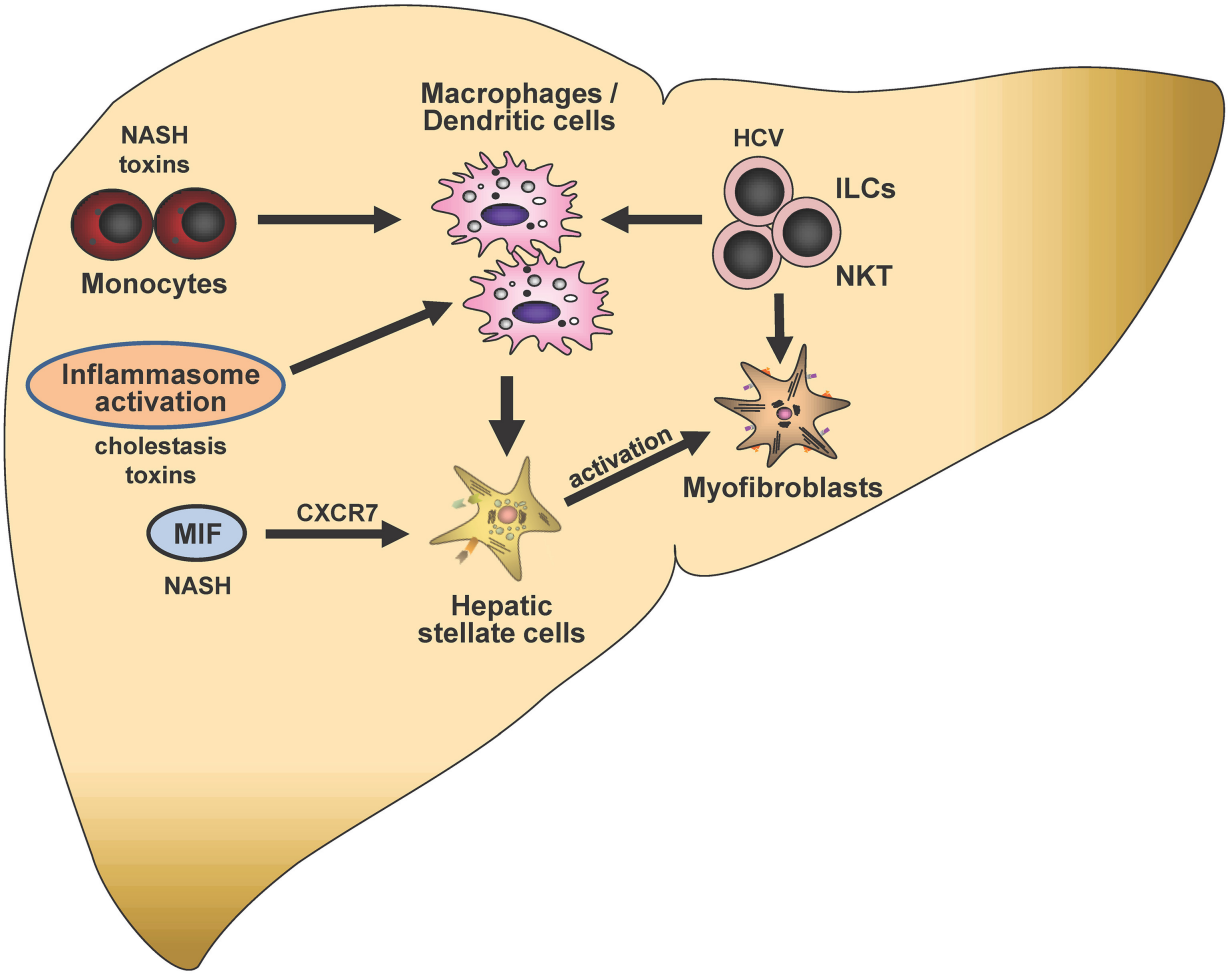
Immunological mechanisms in liver fibrogenesis. Selected potential signaling pathways and proposed cell-cell interactions discussed in this article are shown schematically. Mediators and triggers are depicted in smaller font size as follows: NASH, non-alcoholic steatohepatitis; CXCR7, CXC chemokine receptor type 7; HCV, hepatitis C virus; ILCs, Innate lymphoid cells; NKT, Natural killer T-cells; MIF, Macrophage migration inhibitory factor. In the center of this chapter is the potential crosstalk of monocytes, macrophages, hepatic stellate cells and ILCs, which can be modulated by distinct triggers such as NASH, toxins, inflammasome activation or HCV infection. Crosstalks may eventually lead to modulation of stellate cell activation and subsequent liver fibrogenesis.

Understanding immunological mechanisms is central for the research and therapy of liver fibrosis. Here, we will review selected approaches designed to clarify immunological mechanisms that drive the initiation, progression and regression of fibrosis in the liver as illustrated in Figure 5. Areas of interest included—among others- the identity of the pro-fibrotic immune cells infiltrating the liver, the chemokines that attracted them, their interaction with tissue-resident extracellular matrix (ECM)-producing cells as well as mechanisms of fibrosis resolution as specified in detail as follows.

### Targeting MIF Family Proteins in Liver Fibrosis

Macrophage migration inhibitory factor (MIF) is an evolutionarily conserved defense protein and upstream regulator of innate immunity that was more recently re-defined as a broadly expressed and pleiotropic inflammatory cytokine. The 3D architecture of the MIF trimer is similar to tautomerase enzymes found in various kingdoms, but the MIF monomer also exhibits similarities to dimeric CXC chemokines such as CXCL8. In fact, MIF engages in high-affinity non-cognate interactions with the CXC chemokine receptors CXCR2 and CXCR4 and accordingly, has been recognized to be a prototypical member of the emerging family of atypical chemokine (ACK) (77–79). MIF promotes monocyte and neutrophil recruitment through CXCR2, while enhancing T-cell, B-cell, and progenitor cell recruitment, as well as cancer cell metastasis through CXCR4. Combined activation of the MIF/CXCR2 and MIF/CXCR4 axes drives monocyte/macrophage atherogenic recruitment and is a major driver of atherosclerotic lesion development (78, 80). In addition to the CXC chemokine receptors, MIF interacts with CD74, the membrane form of invariant chain (Ii). While Ii functions as an MHC class II chaperone with a critical role in class II trafficking and control of peptide loading in the endolysosomal compartment, the membrane form of CD74 acts as cytokine receptor for MIF (81). Under inflammatory and neoplastic conditions, CD74 is upregulated also in the absence of class II (82). Receptor activities of CD74 encompass proliferation and survival responses through NF-κB/TAp63 and ERK-MAPK, while blocking p53-mediated apoptosis (82). Signaling through MIF/CD74 requires recruitment of signaling-competent co-receptors, which can either be the accessory protein CD44 mediating Src tyrosine kinase and PI3K/AKT activation, MIF associated chemokine receptors, which can form hetero-oligomeric complexes with CD74 (78), or nuclear translocation of the intracellular domain (ICD) of CD74. The more recently described MIF homolog D-dopachrome tautomerase (D-DT/MIF-2) shares an inflammatory activity spectrum with MIF in sepsis, but has opposing properties in adipose inflammation, and also engages the MIF cognate receptor CD74 (83, 84).

MIF is a critical mediator in the pathogenesis of various inflammatory and immune diseases such as septic shock, rheumatoid arthritis, and atherosclerosis, as well as several cancers (80, 85). MIF levels correlate with the inflammatory status and disease stage and the predominant MIF gene promoter polymorphisms, a −173 G/C SNP and −794 (CATT)_5−8_ microsatellite repeat, are associated with the severity of asthma, atherosclerosis, kidney injury, and rheumatoid arthritis (86, 87). With regard to fibrotic disorders, MIF has been firmly linked to cystic fibrosis, fibrosis in the bladder, and myocardial interstitial fibrosis (88, 89). Here, we discuss current knowledge on MIF's role in liver disease and fibrogenesis.

In the liver, MIF is not only produced by immune, endothelial cells, and cancer cells, but also by hepatocytes. In a study on alcoholic liver injury, MIF serum levels were significantly augmented in patients with alcoholic steatohepatitis (ASH) and alcoholic cirrhosis as compared to controls and MIF expression in ASH could be pinpointed to (ballooned) hepatocytes and infiltrating inflammatory cells i.e., neutrophils (90). This data is supported by a recent study identifying the liver—and more specifically hepatocytes—as significant source of circulating MIF levels in ASH, which correlated with disease severity and mortality (91). On the other hand, one study analyzing MIF expression and the MIF gene −173 G/C polymorphism in NASH failed to detect a correlation between MIF expression in hepatocytes and fibrosis stage, but observed that MIF expression of mononuclear cells in liver tissue significantly increased according to fibrosis stage.

Besides metabolic liver injury, MIF has also been functionally implicated in the course of human chronic liver diseases of various etiologies such as autoimmune disorders or chronic viral infections (92–94). In HCV-induced liver fibrosis, microsatellite polymorphism −794CATT_5−8_ and the −173G/C SNP were predictive of fibrosis severity and cirrhosis-associated complications such as impaired liver function and prevalence of hepatocellular carcinoma (94). Moreover, MIF promoter polymorphisms and serum levels in patients correlated with the presence of autoimmune hepatitis and primary biliary cholangitis (92, 93).

Functional studies in two independent mouse models of hepatotoxin-driven chronic liver injury revealed a protective effect of MIF, which could be attributed to inhibitory effects on hepatic stellate cell activation and proliferation—key events in liver fibrogenesis—via engaging the CD74/AMPK pathway (95). Moreover, MIF/CD74/AMPK signaling in hepatocytes prevented fatty degeneration in a model of NASH (96). The activation of these protective pathways in the liver is reminiscent of reported cardioprotective activities of MIF mediated through the CD74/AMPK axis (80). In experimental models of ethanol-induced liver injury, however, MIF's impact seems to be context-dependent. While MIF exacerbates liver injury during chronic ethanol feeding by modulating chemokine production and immune cell infiltration (91, 97), it mediates protective effects following chronic-binge ethanol feeding by regulating the unfolded protein response in hepatocytes (98). A similar effect might also be seen during the course of NASH. While MIF conveys anti-steatotic effects on hepatocytes, it contributes to liver fibrogenesis in a murine model of NASH (MCD diet feeding) by skewing the intrahepatic immune milieu toward a pro-fibrotic polarization of innate lymphocytes (99).

**Figure 6:**
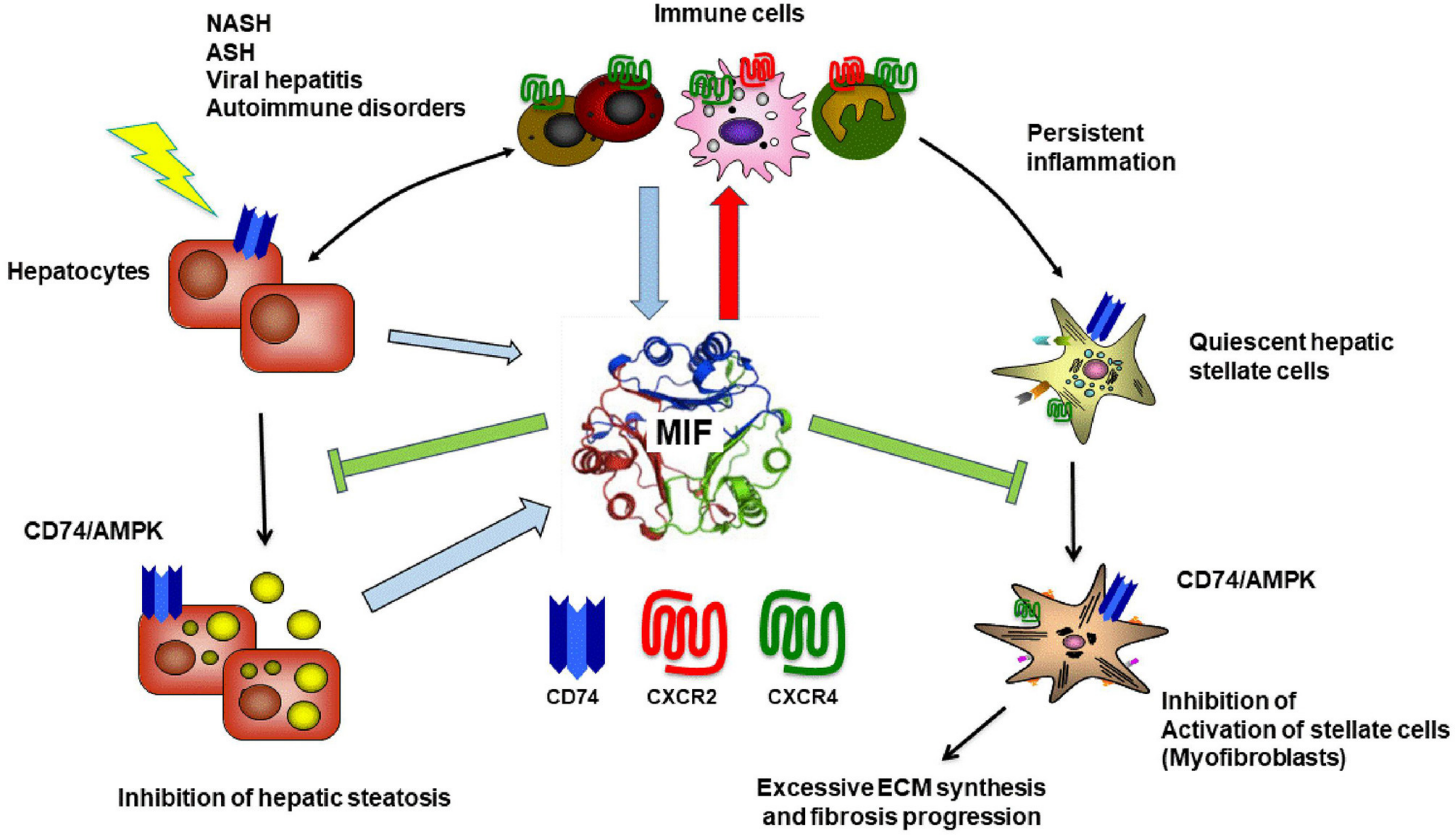
Macrophage migration inhibitory factor in chronic liver injury and fibrogenesis. In the liver, the atypical chemokine Macrophage migration inhibitory factor (MIF) is mostly expressed by infiltrating immune cells as well as hepatocytes especially under stress condition e.g., fatty degeneration or inflammation during NASH, ASH and viral hepatitis. In addition, it is most likely that MIF is involved in the pathogenesis of hepatic autoimmune disorders because this cytokine was previously shown to be associated with many autoimmune disorders including rheumatoid arthritis, inflammatory arthritis, inflammatory bowel disease, multiple sclerosis, autoimmune uveitis., autoimmune glomerulonephritis, systemic lupus erythematosus, sarcoidosis and many other autoimmune inflammatory disorder [for review see (85, 100)]. MIF engages high-affinity non-cognate interactions with three different surface receptors expressed by liver resident cells as well as infiltrating cells, the CXC chemokine receptors CXCR2 and CXCR4 as well as with CD74, the membrane form of invariant chain (Ii). While MIF engagement with the CD74 receptor on hepatocytes and hepatic stellate cells exert protective effect via AMPK signaling during chronic liver injury, MIF also mediates pro-inflammatory effects orchestrating the intrahepatic recruitment and activation of inflammatory immune cells via engagement of CXCR2 and CXCR4. The balance between these opposing, regulatory roles of the MIF/receptor network is crucial for the overall impact of MIF on severity and progression of chronic liver disease in distinct settings and has to be considered when designing MIF-directed therapeutic strategies. Blue arrow indicates MIF release. Red arrow marks pro-inflammatory properties and green arrow protective features of MIF on respective pathways.

In summary, these results underline a pivotal, but complex role of MIF during chronic liver diseases, with even dichotomic effects in response to the same insults. Figure 6 summarizes the regulatory role of the MIF/receptor network in chronic liver diseases. Considering the complexity of this network is of specific importance when designing MIF-directed therapeutic strategies in the setting of chronic liver disease. Of note, several efforts to therapeutically target the MIF system are currently assessed in early clinical trials concerning various disease settings such as rheumatic, cardiovascular disease, or neoplastic disorders, including small molecule-, antibody- and peptide-based approaches targeting either MIF or specific MIF receptor-mediated pathways (80). Based on the mechanistic insight from the experimental data, one might speculate that a key determinant of MIF-mediated outcome could be the predominance of MIF's impact on immune cell recruitment/polarization over its direct effects on liver resident cells. This predominance might be guided by specific intrahepatic MIF receptor expression patterns. Whether these patterns are a valid and stable surrogate to predict MIF-mediated effects warrants further investigations, which also should consider potential distinct activities of D-DT/MIF-2. A further comprehensive understanding of MIF's role in the different settings and even at distinct disease stage is a prerequisite to guide MIF-directed therapeutic interventions and predict the outcome of these therapeutic strategies in clinical practice during the course of chronic liver injury.

### Monocyte and Macrophages in Liver Fibrosis

Macrophages comprise the most abundant immune cell type of the liver, playing an essential part in the maintenance of liver homeostasis and in reparative or propagating mechanisms following acute or chronic liver injury. Two major subtypes of hepatic macrophages can be distinguished; the liver resident Kupffer cells (KCs), which are considered to be a self-sustaining and often tolerogenic phagocyte population, and the immunogenic monocyte-derived infiltrating macrophages (MoMΦs) (101, 102). Interestingly, upon liver disease, dynamic changes in these macrophage subsets can be observed, such as a rapid loss of KCs after injury, and an increased recruitment and subsequent accumulation of inflammatory MoMΦs that are characterized by Ly6C^high^ (Gr-1) expression in mice. Moreover, such infiltrated Ly6C^high^ MoMΦs, of which the recruitment was shown to be critically controlled by the chemokine CCL2/CCR2 signaling pathway, represent the dominant macrophage population during the early phases of liver injury, promoting the progression of liver fibrosis by activating hepatic stellate cells (HSCs) and other myofibroblasts (103). Furthermore, the pharmacological inhibition of CCL2 with the Spiegelmer-based antagonist mNOX-E36 efficiently inhibits the infiltration of Ly6C^high^ MoMΦs into chronic CCl_4_- or methionine-choline-deficient (MCD)-diet-induced injured liver in mice, ameliorating hepatic inflammation and steatosis (104). Administration of mNOX-E36 during the regression phase of these murine models of liver disease, however, significantly accelerated fibrosis resolution, by causing a shift in hepatic dominance from the pro-inflammatory, tumor necrosis factor (TNF)-secreting, Ly6C^high^ MoMΦs toward “restorative” Ly6C^low^ MoMΦs (105). Also cenicriviroc (CVC), an orally available dual CCR2/CCR5-inhibitor has been shown to efficiently block monocyte recruitment, and to promote amelioration of insulin resistance as well as anti-inflammatory-, and anti-fibrotic effects in murine NASH models (106). This drug has been evaluated in a large (*n* = 289 patients) biopsy-controlled phase 2b clinical trial in NASH patients, in which CVC promoted accelerated fibrosis regression after 1 year of therapy (107). However, the anti-fibrotic efficacy appears neither increased nor sustained over 2 years of therapy (108). CVC had entered phase 3 clinical development with about 2,000 patients (109), but the trial was prematurely terminated since the primary endpoint of fibrosis improvement after 1 year was not reached upon pre-specified interim analysis.

Besides the CCL2/CCR2-pathway, also the CCL1/CCR8-signaling cascade represents an important chemoattraction axis. Indeed, *Ccr8*^−/−^ mice undergoing chronic CCl_4_-injections or bile duct ligation (BDL) display reduced infiltration of inflammatory MoMΦs, neutrophils and natural killer (NK) cells, whereas the number of hepatic CD4+ T cells was increased. An overall protection from liver fibrosis was observed in these models in *Ccr8*^−/−^ mouse, when compared to their respective controls (110). The importance of chemokine-axes can be further demonstrated by the regulation of hepatic inflammation through the CXCR6/CXCL16-axis during liver disease. CXCL16 was found to be secreted by hepatic endothelium and macrophages. It can control the recruitment and functionality of CXCR6-expressing pro-inflammatory NK T-cells, especially during the early response in experimental liver damage. Moreover, reduced NK T-cell accumulation in *Cxcr6*^−/−^ mice led to the diminished presence of the pro-inflammatory cytokines IFN-γ, TNF-α, and IL-4 in the hepatic microenvironment, and overall reduced fibrogenesis (111).

**Figure 7:**
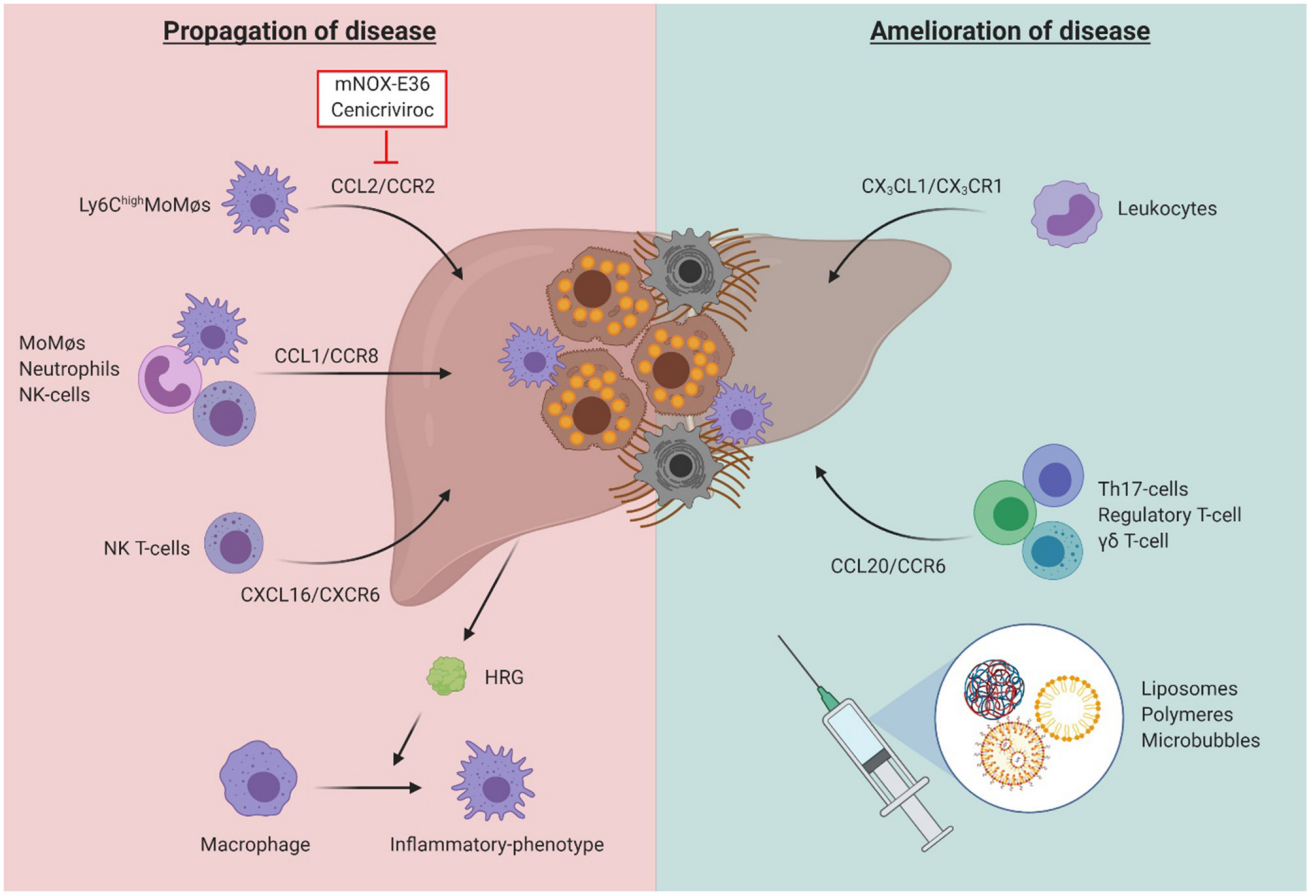
The dual role of inflammatory cells in liver fibrosis. While the chemokine-axes CCL2/CCR2, CCL1/CCR8, and CXCL16/CXCR6 have been shown to play a role in disease propagation through induction of a pro-inflammatory and pro-fibrogenic environment, the axes CX_3_CL1/CX_3_CR1, and CCL20/CCR6 were identified to be essential to obtain amelioration of liver function after acute or chronic damage. Besides its effects on inflammatory cell recruitment, the liver environment also influences the function of immune cells, such as through secretion of histidine-rich glycoprotein (HRG), promoting the polarization of macrophages toward an inflammatory phenotype. While therapeutic agents targeting the inflammatory system may be a promising strategy, their potential off-target effects limit their future use. Cell-specific nano-scale delivery systems such as liposomes, polymers and microbubbles may therefore aid in the development of such inflammatory-specific therapeutic tools.

While all above-mentioned research suggests the interception of chemokine-axes as potential therapeutic strategy, it should be noted that the inflammatory system has a dual function in liver disease, and that some chemoattractants may thus be necessary for disease amelioration and tissue repair (Figure 7). For example, CX_3_CL1 (also known as fractalkine) is shed by HSCs and hepatocytes upon liver injury, creating a chemo-attractive gradient for the CX_3_CR1-expressing leukocytes. Surprisingly, the abrogation of this axis in CCl_4_- or BDL-challenged mice causes an increased presence of pro-inflammatory TNF/iNOS- producing MoMΦs and subsequent extent of fibrosis (112). The CCR6/CCL20-axis has a similar –protective—functionality. Upon liver damage, the secretion of CCL20 by parenchymal cells is elevated, causing increased recruitment of CCR6-expressing T-helper (Th)17, regulatory, and gamma-delta (γδ) T-cells. Elimination of this signaling cascade during liver disease caused by CCl_4_ and MCD-diet, using mice with a *Ccr6*^−/−^ phenotype, identified reduced accumulation of interleukin (IL)-17 and −22 expressing γδ T-cells, and causing more inflammation and fibrosis as compared to wild type mice. These effects would be partly derived through the potential of γδ T-cells to induced HSC apoptosis in a cell-cell contact dependent manner involving Fas-ligand (CD95L) (113).

The injured liver environment not only tightly regulates the inflammatory response through secretion of chemoattractants, and thus the recruitment of inflammatory cells, but also by shaping the inflammatory cell polarization and functionality. One example includes histidine-rich glycoprotein (HRG), a liver-derived plasma protein, which promotes the polarization of macrophages toward the proinflammatory (“M1-like”) phenotype, thus stimulating the propagation of liver injury and subsequent fibrogenesis (114). Disease-induced effects on immune cell function and polarization are even suggested to be obtained in the precursory bone marrow monocytes, and remain conserved in their functional derivates, the hepatic myeloid cells. For example, in murine models of NAFLD, a prominent down-regulation of calprotectin coding genes *S100a8* and *S100a9* is observed in the myeloid cells of both the liver and bone marrow (115). Peroxisome proliferator-activated receptors (PPARs) are nuclear receptors, subdivided into 3 isoforms (PPARα, PPARγ, and PPARδ/β), with PPARγ and PPARδ being involved in macrophage polarization. Such effects where proven through administration of lanifibranor, a pan-PPAR agonist, to palmitic acid-stimulated murine macrophages and patient-derived circulating monocytes, leading to a reduction in the expression of proinflammatory genes. When administered to choline-deficient amino acid-defined high fat (CDAA)- and Western-diet (WD) mice models, lanifibranor showed significant beneficial effects on the extent of steatosis and hepatitis, thus suggesting its potential use as therapeutic agent for NAFLD/NASH (116).

While modulators of immune cell recruitment, functionality and polarization may represent interesting therapeutic options in the battle against liver disease, their systemic administration may cause unwanted side-effects. Nanomedicine-based approaches, targeting specific cell types, may eliminate this drawback. Different nano- and micrometer-sized drug delivery systems have been developed and were shown to target distinct cellular players of the inflammatory process (117). Indeed, after systemic administration, PEGylated liposomes are rapidly taken up by the dendritic cells of the liver, lung, and kidney, poly(N-(2-hydroxypropyl) methacrylamide) polymers by the endothelial cells in the liver, neutrophils and alveolar macrophages, and poly n-butylcyanoacrylate (PBCA) microbubbles by KC and splenic red pulp macrophages (118). The absence of any effects of these drug carrier systems with regard to hepatotoxicity or inflammation, regardless of its efficient uptake by myeloid immune cell in the liver, is promising concerning future clinical application (119). It should be noted that the fibrotic liver environment significantly affects the targeting efficiency of the different mentioned carrier systems, therefore suggesting the need for an adapted nanomedicine-based approach for interfering with early events in the pathogenesis of chronic liver disease (120).

### Innate Lymphoid Cells and Liver Fibrosis

Innate lymphoid cells (ILCs) represent a heterogeneous family of innate immune cells with lymphoid phenotypes, but lack rearranged antigen receptors (121). Traditionally, ILCs have been divided into 3 groups based on the expression of specific transcription factors, cell-surface markers, and signature cytokines. Members of group 1 are IFN-γ producing and T-bet dependent ILCs (ILC1s). Group 2 ILCs (ILC2s) are a population of cells that preferentially produce type 2 cytokines, including IL-5 and IL-13, and require GATA3. Group 3 ILCs (ILC3s) can produce IL-17 and/or IL-22, and are dependent on RORγt.

This classification was recently revised. Two new members were added to the ILC family: conventional NK cells (cNK) and lymphoid tissue-inducer cells. However, the unambiguous definition of ILC1 and cNK and their differentiation from each other is still challenging and is likely to differ between mice and men (122). For instance, transcriptional profiling of hepatic ILCs demonstrated mouse liver ILC1s to display unique expression patterns for several chemokine receptors and adhesion molecules, including CXCR6, CD103, CD49a, CD69 (123, 124), markers that have been proposed to characterize liver resident NK (lrNK) cells in humans (125, 126). Within each group, ILCs are heterogeneous in terms of phenotype and cytokine profiles within tissue and between different organs, both in mice and humans. Moreover, there is increasing data suggesting that localization of ILC subsets in specific compartments relates to their roles in immune and inflammatory responses.

cNK cells are critically involved in the immune-pathogenesis of liver disease and both murine and human cNK cells have been shown to exhibit anti-fibrotic activity by the induction of apoptosis and/or killing of activated star cells (127, 128). This anti-fibrotic function of cNK cells is linked to the surface expression of activating NK cell receptors including NKG2D, NKp46, and NKp30, that recognize specific molecules expressed on activated HSC. In addition to NK cell receptors, members of the TNF superfamily are also involved in the anti-fibrotic activity of NK cells. Activation of HSCs leads to an increased expression of TRAIL receptors resulting in an increased susceptibility to apoptosis induction by TRAIL-expressing NK cells (128).

Of note, most of these data were obtained in studies with peripheral cNK cells, so it is unclear to what extent these results can be extrapolated to lrNK cells. In addition, further work is needed to clarify which subsets of lrNK cells are involved in modulating liver fibrosis. Moreover, it is important that the function of NK cells is influenced by the surrounding microenvironment, such as local cytokine concentration or interactions with other immunocompetent cells. For instance, we demonstrated that CD4^+^ T cells effectively trigger anti-fibrotic cNK cell activity in an IL-2 dependent fashion (129) whereas regulatory T cells can exert inhibitory effects on NK cell anti-fibrotic activity, thus promoting fibrogenesis (130). In addition, chronic liver cell damage leads to increased levels of Transforming growth factor-β (TGF-β), which inhibits the anti-fibrotic function of NK cells by downregulating NKG2D and 2B4. With regard to the other members of the ILC family, data on their possible role in hepatic fibrogenesis are rather sparse. McHedlidze et al. demonstrated that in mice interleukin-33 (IL-33), secreted by injured hepatocytes, activates ILC2 to produce IL-13, which then induces activation of hepatic stellate cells, thereby promoting hepatic fibrogenesis (131). This observation provided first evidence that ILCs other than cNK cells also modulate hepatic fibrosis.

In the context of human liver disease, Forkel and co-workers observed a correlation between the severity of fibrosis and the proportion of intrahepatic ILC2, which may produce IL-13 and mediate pro-fibrotic activity. The increased production of IL-33 and thymic stromal lymphopoeitin by hepatocytes, HSCs and Kupffer cells is discussed as a possible mechanism of ILC2 activation (132). However, given the low abundance of ILC2 in the human liver the exact role of this subset still has still to be defined. With respect to ILC3, Wang et al. observed an intrahepatic accumulation of IL-17 and IL-22 producing ILC3, which displayed pro-fibrotic activity in CCl_4_-induced liver fibrosis (133). Whether human group 3 ILCs also play a role in hepatic fibrogenesis is unclear at the moment. However, given their important role in maintaining intestinal health by promoting immunity to pathogens, limiting inappropriate inflammatory responses to commensal bacteria or dietary antigens, or mediating repair following tissue damage ILCs may also indirectly modulate liver fibrosis via affecting the so called “gut-liver axis.” Both, derangement of the gut microflora (the microbiome) as well as defects of the intestinal barrier integrity have been shown to promote microbial translocation (MT) and there is accumulating evidence, mainly obtained in mouse models, indicating that ILCs critically affect both parameters. Exposure of hepatic immune cells to such gut-derived microbial products is considered to trigger hepatic inflammation in an inflammasome-dependent manner and to modify immune responses of intrahepatic immune cells, thereby accelerating hepatic fibrogenesis. Thus, it is tempting to speculate that alterations of the intestinal ILC population in liver disease may result in increased microbial translocation, thereby promoting hepatic inflammation and fibrogenesis.

Despite relevant progress, our understanding of hepatic ILCs and their role in fibrogenesis is still incomplete. Important questions concerning the role of specific subsets, the influence of the hepatic microenvironment and the gut-liver axis still need to be answered in detail to improve our understanding of the immunopathogenesis of liver cirrhosis.

### NLRP3 Inflammasome Activation in Liver Disease Progression

Inflammasomes are intracellular multi-protein complexes expressed in parenchymal as well as non-parenchymal cells in the liver. They act as key regulators of inflammation and cell fate in various diseases (134). In humans, *Nlrp3* gain-of-function mutations are causing a spectrum of rare auto-inflammatory disorders known as cryopyrin associated periodic syndromes (CAPS). The nucleotide-binding oligomerization domain-like receptor (NLR) pyrin domain containing 3 (NLRP3) inflammasome consists of NLRP3, apoptosis-associated speck-like protein containing a caspase recruitment domain (ASC), and pro-caspase-1. The assembly of these three main components is triggered by different molecular and pathogenic structures such as bacterial LPS, adenosine triphosphate (ATP), uric acid, reactive oxygen species (ROS), fatty acids, bile acids (134). The activation of the functional inflammasome requires two signals (or two hits), one for inducing gene expression and assembly and the second for activating the effector component caspase 1. Active caspase-1 mediates the cleavage and release of the pro-inflammatory cytokines IL-1β and IL-18. Alongside *Nlrp3-*associated programmed cell death termed pyroptosis is initiated ultimately causing cell swelling and disruption of plasma membrane due to the assembly of the pore forming gasdermin D. This form of cell death is triggered by pro-inflammatory signals and associated with inflammation in which processes are triggered that enhance or initiate attraction and activation of immune cells but also perpetuate an abnormal wound-healing response (135, 136). This is also reflected in the term pyroptosis composed of “pyro” and “ptosis.” While “pyro” comes from the Greek word fire indicating the properties of an inflammatory reaction, the Greek word term “ptosis” standing for falling indicate the processes that are associated with the process of cell death (136).

Methionine- and choline-deficient diet (MCDD) or prolonged high fat diet (HFD) induced murine NASH characterized by steatosis and immune cell infiltrates, display increased hepatic mRNA expression of IL-1β, NLRP3, caspase 1, and ASC alongside elevated caspase-1 activity (137, 138). NLRP3 knockout mice fed a choline-deficient amino acid-defined [CDAA] diet were protected from similar inflammation and fibrosis development (139). Hepatic stellate cell (HSC) specific NLRP3-inflamasome-activation induced transdifferentiation to pro-fibrotic myofibroblasts, with livers of 24 weeks old mice showing increased expression of fibrotic α-smooth muscle actin (α-SMA) and collagen independent of inflammation (140). Interestingly, severe inflammatory changes associated with universal overactive *Nlrp3* were almost completely rescued by TNF knockout alongside decreased IL-1β levels, while IL-17 deletions had only minor influence on the induced phenotype (141). Specific blocking of TNF by eternacept also rescued the gain of function phenotype, while reducing serum IL-1ß and IL-18 levels significantly *in vitro* and *in vivo* (142). Targeting the inflammasome with the selective small-molecule inhibitor MCC950 suppressed infiltration with immune cells in NASH caused by overnutrition in atherogenic diet-fed foz/foz mouse model alongside decreased IL-1β, IL-6, and MCP-1 levels (143). These results warrant targeting *Nlrp3* as a possible therapeutic approach. In addition to its effect on the liver, global *Nlrp3* knockout mice fed with MCDD for 3 weeks showed hepatic steatosis alongside alterations in the gut microbiota. Co-housing resulted in exacerbated fatty liver phenotype in wild type animals (144). In the MDR2 knockout model of primary sclerosing cholangitis, intestinal dybiosis was associated with pronounced *Nlrp3* inflammasome activation in the gut-liver axis. Microbiome transfer from healthy mice markedly reduced liver injury in the recipient, so did the pan caspase inhibitor IDN-7314 hinting toward the importance of caspase activation to promote liver injury (145). *Nlrp3*-deficient mice that underwent bile duct ligation (BDL) as a model for primary sclerosing cholangitis (PSC) had significantly less inflammation in acute (2 days) and chronic (28 days) injury in liver and kidney, hinting toward an important role of the inflammasome as well. This finding was confirmed with the use of the specific *Nlrp3* inhibitor MCC950 that cut down disease progression in wild type mice in the BDL model (146).

**Figure 8:**
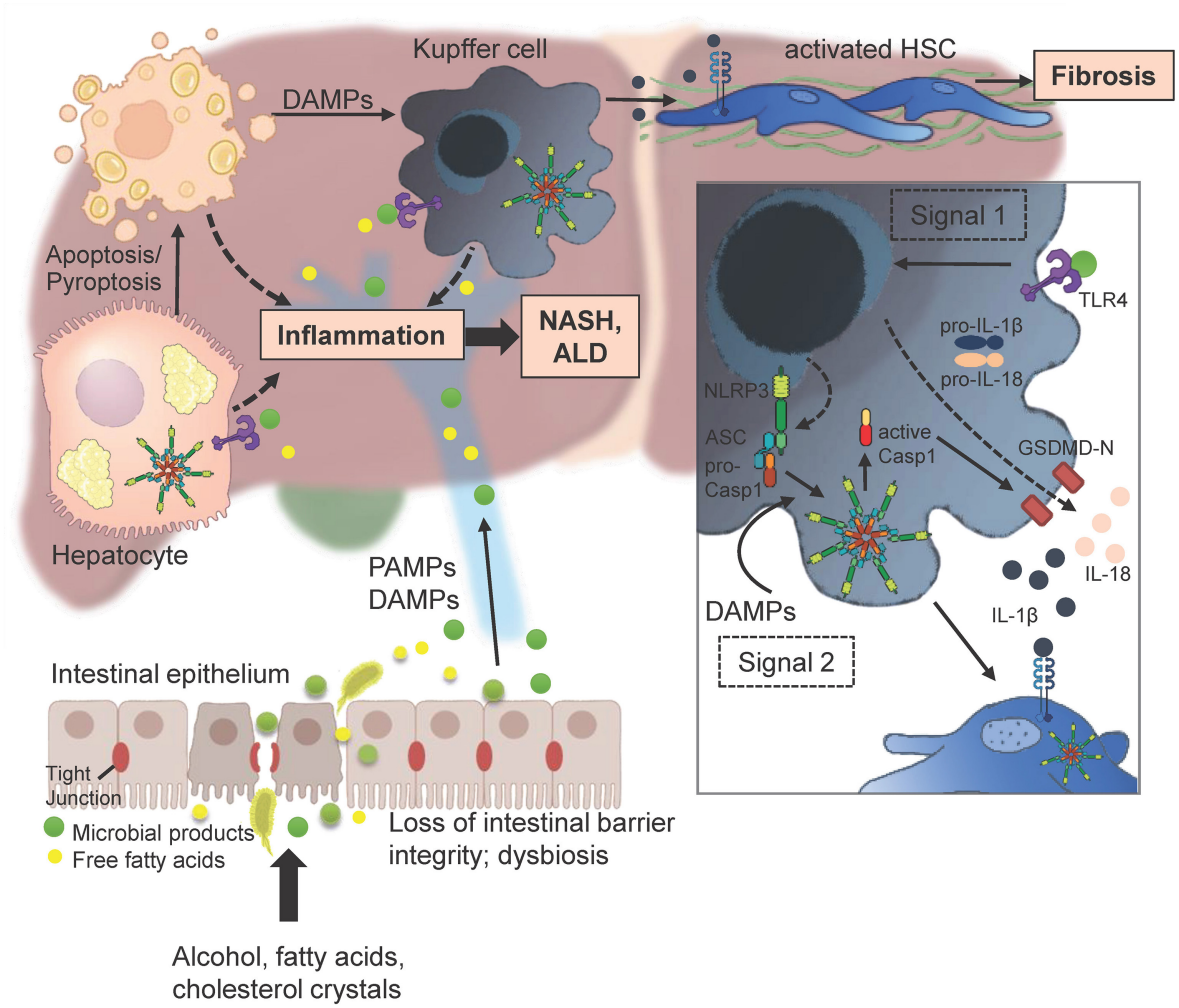
Multitudes of metabolic products like alcohol and free fatty acids can lead to enhanced intestinal permeability by disrupting tight junctions of intestinal epithelial cells. PAMPs and DAMPs that enter the liver initiate gene transcription of pro-IL-1β, pro-IL-18, and NLRP3 itself by binding to an appropriate receptor (here TLR4; signal 1). Injured and dying hepatocytes release DAMPs including endogenous ATP or uric acid that promote the assembly of the three main effectors NLRP3, ASC and procaspase 1 to form the inflammasome. The active caspase 1 cleaves pro-IL-1β and pro-IL-18 as well as Gasdermin (GSDMD) into their mature forms. The N-terminal GSDMD fragments form a membrane pore to enable the release of IL-1β and IL-18 in order to attract further immune cells (Figure was partly created by Biorender).

Recently, a lot of effort was focused on the evaluation of gut microbiota, which was found to be important in the constant interplay between gut and liver. NASH condition induced by MCD diet was markedly improved using the depletion of gut microbiota and consequent repopulation. Commensal microbiota appears to be hepatoprotective in that regard (147). Gastrointestinal dysbiosis associates with increased production of PAMPs and DAMPs, which then enter portal circulation and promote NLRP3 inflammasome activation and specifically inflammation in the liver most importantly by interacting with TLR4 (Figure 8) (148). In obesity, increased intestinal permeability attenuated the expression of tight junctions, which facilitated the effect (149). Depletion of the G-Protein coupled receptor CX3CR1 was associated with significantly altered intestinal microbiota composition due to an impaired intestinal barrier. Endotoxin levels in portal serum and consequently inflammatory macrophages in liver were increased in CX3CR1 deficient mice, indicating an increased inflammatory response (150). As persistent inflammation of gut and fibrosis are preconditions for the development of hepatocellular carcinoma (HCC), effort focused on connecting inflammation in the liver with tumor biology. Signaling of Toll like receptors through the universal adaptor Myd88 and consequent IRAK resulting in NF-κB activation might be important in this process. Functional evaluation of the CC-chemokine ligand 5 in murine model of HCC using hepatocyte specific knockout of NF-κB essential modulator (NEMO) reduced TNF induced apoptosis in those mice, alongside reduced immune cell infiltration of granulocytes and pro inflammatory monocytes (151). While hepatocyte-specific deletion of MyD88/NEMO promotes the tumor progression in mice, this development was ablated in global Myd88/hepatocyte-specific knockouts of NEMO providing further evidence that inflammatory signaling through TLR in non- parenchymal cells of the liver is a driver of disease progression and might be a relevant target for disease treatment or prevention (152).

In summary (see Figure 8), the Nlrp3 inflammasome was shown to be important in fibrosis progression in various models of murine liver disease. Especially continuous inflammatory conditions are often causing for advanced stages of liver fibrosis including HCC. Hence, targeting inflammation and especially NLRP3 seems a viable choice in decreasing severity and slowing fibrosis progression in the future.

### Impact of Chronic IFN-I Signaling in Liver Fibrosis on Anti-viral Immunity

Next to its metabolic functions, the liver represents an important immunological organ housing the largest macrophage population in the body, the Kupffer cells, as well as a great number of conventional and innate-like lymphocytes. Due to its anatomical location, the liver with its arsenal of immune cells acts as firewall against invading pathogens as well as microbial products crossing the gut barrier (153).

Chronic liver injury, caused by toxins, viral infections or auto-immune disease can lead to the development of liver fibrosis and cirrhosis, resulting in a progressive loss of functional liver parenchyma as well as an impairment of the antimicrobial functions of the liver. The gut-liver axis plays a pivotal role in the perpetuation and outcome of liver fibrosis, as the loss of barrier integrity and translocation of gut microbiota have emerged as key risk factors associated with the high incidences of bacterial infections, the aggravation of the fibrotic process and long-term malignancy and HCC development (154, 155). Acute bacterial and viral infections leading to hepatic decompensation and multi-organ failure account for the most important clinical consequences and constitute the main cause of morbidity and mortality in patients with liver cirrhosis (156–158). Previous data suggested that the enhanced susceptibility to infection and overall suboptimal immune responses were due to impaired innate immune cell functionality, as impairments in macrophage (159) and neutrophil function as well as defects in the complement system (160) were reported. The exact mechanisms determining the failure of cirrhotic patients to contain bacterial infections however, remained unclear.

Within the SFB/TRR57 consortium, it has been discovered that innate sensing of translocated gut microbiota by hepatic myeloid cells induces a chronic production of type I interferon that massively impairs innate immune responses to *de novo* bacterial infections during cirrhosis (161). Upon infection with cytosolic pathogens such as *Listeria*, IFNAR signaling in these myeloid cells triggers the expression of IL-10, resulting in the inhibition of key bactericidal mechanisms in monocytes and macrophages (162). Using two different mouse models of liver fibrosis, i.e., BDL and CCl_4_ injection, it was shown that liver fibrosis is associated with enhanced lethality and impaired clearance of the pathogenic bacteria *Listeria monoytogenes*. Beside impaired phagocytosis and reduced production of effector cytokines such as IL-12 and IL-1β, the chronic IFNAR signaling in mice with fibrosis led to impaired granulopoiesis indicated by drop of neutrophil numbers in the blood upon *Listeria* infection. Using Germ-free or TLR-deficient mice it was shown that TLR-mediated sensing of microbiome-derived PAMPs is the trigger of the “immune suppressive” IFN-I, as myeloid cells from GF mice compared to their counterparts from SPF mice showed normal phagocytic and antibacterial functions as well as low levels of IL-10 upon *Listeria* infection despite liver fibrosis. Importantly, these findings were mirrored in human tissues and cells, as patients with NASH or ASH induced cirrhosis showed significantly higher hepatic IFNβ expression. Moreover, IL-10 production in monocytes was increased after infection with the intracellular bacteria, *L. monocytogenes, Legionella pneumophila, Mycobacterium avium*, or *Salmonella typhimurium*.

Both genetic ablation as well as antibody-mediated inhibition of IFNAR signaling led to improved survival and bacterial clearance in fibrotic mice, suggesting that the described signaling axis could be targeted for the benefit of liver fibrosis patients. Improved anti-bacterial immune response upon inhibition of IFNAR signaling was associated with decreased levels of IL-10, strengthening the idea that IL-10 might mediate the downstream mechanism by which IFN-I exerts its immune suppressive function in fibrosis. Strikingly, antibody-mediated neutralization IL-10 receptor promoted *Listeria* clearance and prevented infection-associated mortality. Of note, blockade of IL-10R signaling in the fibrotic mice after Listeria infection did not induced any immune pathology in our models. These findings suggested that interference with the IL-10R signaling pathway in patients with liver cirrhosis might offer a safe therapeutic option with beneficial effects on antibacterial immune defenses and reduce infection associated mortality.

These results highlight the pathophysiological importance of gut microbial translocation in liver cirrhosis and identify IL-10 and interferon receptor signaling as molecular targets for therapeutic intervention to overcome the failure to control infection with intracellular bacteria. The findings further support the key role of the liver as a firewall of the immune system as the loss of this line of defense during liver cirrhosis leads to systemic immune failure upon bacterial infections.

## Repair and Modulation of Liver Fibrosis

**Figure 9:**
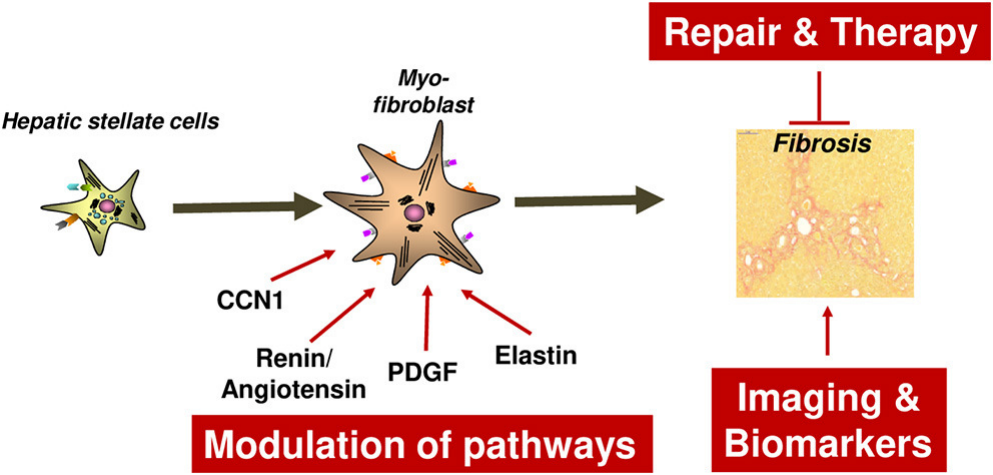
Concepts for the repair and modulation of liver fibrosis. Detailed explanations are given in the main text. CCN1, Cellular Communication network factor 1; PDGF, Platelet-derived growth factor.

In the subsequent paragraphs we will review a selection of novel regulatory mechanisms contributing to the pathogenesis of liver fibrosis that have been investigated in detail by the SFB/TRR57 consortium. In particular, individual members of the CCN protein family representing matricellular proteins that coordinate and promote signaling among extracellular matrix, secreted proteins and cell surface receptors were in the focus of research as illustrated in Figure 9. Similarly, the consortium investigated the influence of the renin-angiotensin system and its counteracting receptors for hepatic fibrosis and portal hypertension. These studies were complemented with the establishment of novel imaging probes and protocols for monitoring, molecular diagnosis, staging and monitoring fibrosis reversal in the liver during pharmacological intervention. As such, this subject area significantly contributed to the understanding of molecular mechanisms of fibrosis and provided a step forward to translate research findings on organ fibrosis to novel diagnostic and therapeutic strategies for future clinical management of patients suffering from organ fibrosis.

### Targeting CCN1/CYR61 as a New Treatment Modality in Hepatic Fibrosis

The protein family of Cellular Communication network (CCN) factors contains six matricellular proteins (CCN1-CCN6). A CCN protein is composed of a signal sequence and four distinct structural modules including an insulin-like growth factor binding domain, a von Willebrand factor type C motif, a thrombospondin type I module, and a carboxyl-terminal cystine knot motif containing two disulfide bridges (163–165). These proteins are critically involved in the control of development, cell fate, angiogenesis, tumorigenesis, osteogenesis, cell adhesion, mitogenesis, migration, chemotaxis, cell survival, and extracellular matrix production. CCN proteins are further capable to bind pro-fibrogenic cytokines such as TGF-β and the production of reactive oxygen species (ROS) that are both involved in the production of liver damage and initiation of hepatic fibrogenesis. Most strikingly, individual CCN members can mutually inhibit each other's expression and drive opposite effects of biological processes (166, 167). In this dualistic Yin and Yang activity concept, individual CCN proteins are independently acting but interconnected in a regulatory dynamic network that decides about the outcome of both, physiological and pathological processes. In regard to liver fibrosis, the interplay of CCN actions is even more complex and far beyond the complementary nature of Yin and Yang. This was exemplarily documented in a study showing that the adenoviral overexpression of CCN2 suppressed CCN3 expression, while the overexpression of CCN3 as well as the suppression of CCN3 by targeted siRNAs both resulted in enhanced CCN2 expression (168). *In vivo*, the expression of CCN2 and CCN3 are both increased in models of ongoing fibrogenesis. However, the cellular subsets expressing CCN2 or CCN3 are different and are strongly dependent on the model. In the BDL model, CCN3 expression in damaged liver is majorly found in mesenchymal and proliferating bile duct epithelia cells along the fibrotic septa, while CCN3 is predominant in persinusoidal areas peripheral to centrilobular hepatic necrosteatosis in the CCl_4_ model (168). In contrast, CCN2 expression is markedly increased in damaged and cultured hepatocytes, which however do not express CCN3 (168). Artificial overexpression of CCN3 reduced expression of CCN2 in cultured hepatocytes, but failed to reduce liver fibrogenesis in the BDL model (169). Interestingly, adenoviral overexpression of either CCN2 or CCN3 in cultured hepatocytes induced reactive oxygen species formation and activated p38 and JNK pathways, thereby triggering hepatocyte apoptosis (169).

**Figure 10:**
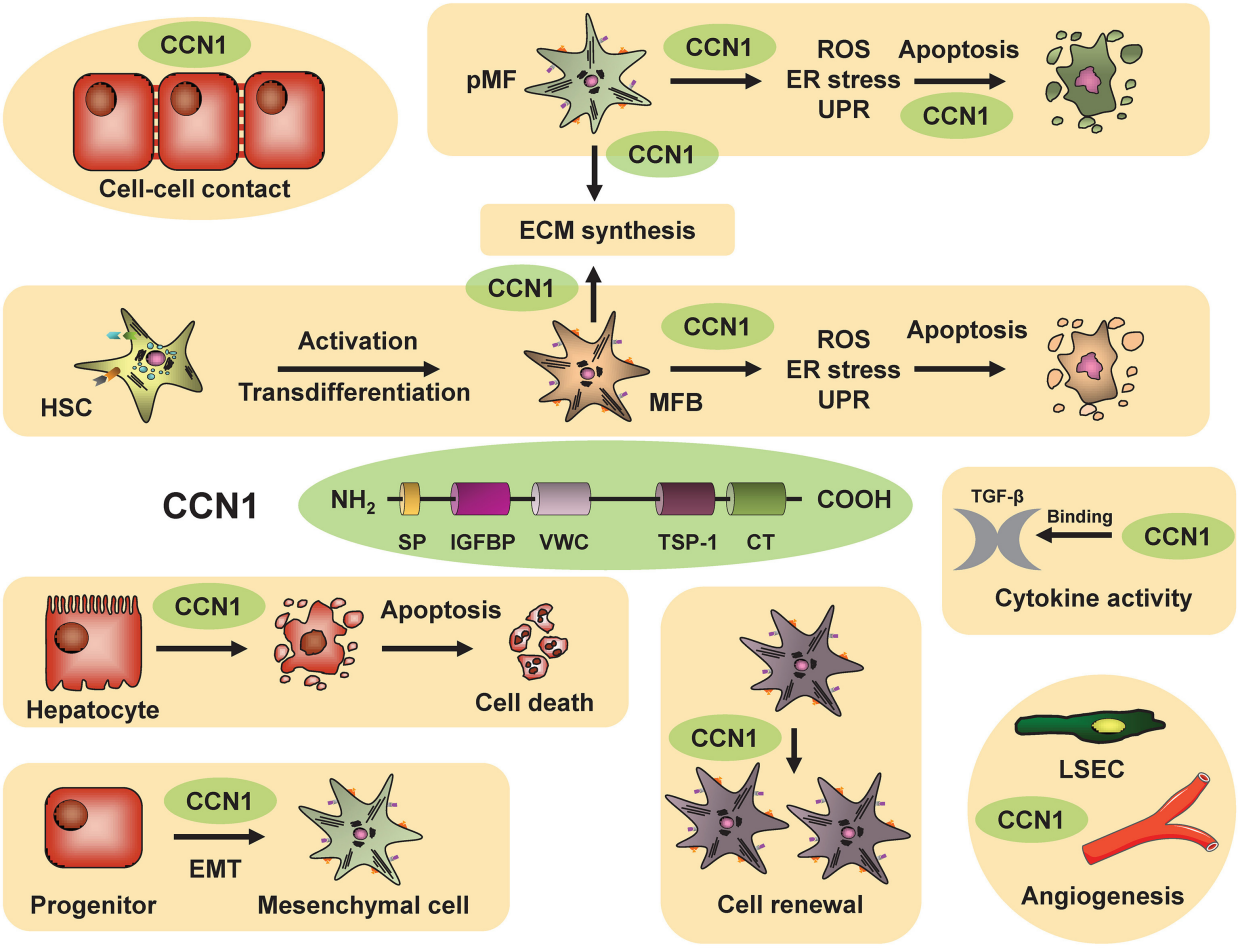
CCN1 in liver homeostasis and disease. CCN1 consists of a secretory signal (SP), an insulin-like growth factor-binding protein domain (IGFBP), a von Willebrand type C domain (VWC), a thrombospondin-1 domain (TSP-1), and a cysteine knot (CT). The biological activities of CCN proteins manifest during liver injury. They stimulate the activation and transdifferentiation of hepatic stellate cells (HSC) to matrix-producing myofibroblasts (MFB), modulate cytokine activity, regulate apoptosis/necrosis, and fine-tune mechanisms involved in control of cell-cell contacts, cell renewal, epithelial-to-mesenchymal transition (EMT), and (neo-)angiogenesis. Large quantities induce endoplasmic reticulum stress and unfolded protein response. Moreover, they can bind cytokines such as TGF-β, thereby modulating their activities and pathways.

In the liver, CCN1 has several important activities (Figure 10). It acts as a senescence inducer that might be particularly important in later stages of wound healing by avoiding progressive fibrosis and by initiating resolution of fibrotic scar tissue (170). When massively overexpressed, CCN1 is directed to the endoplasmic reticulum (ER), resulting in ER stress and unfolded protein response (UPR). The resulting apoptosis of HSC is correlated with reduced collagen expression confirming that CCN1 has the capacity to attenuate liver fibrogenesis by modulating the three phases of endoplasmic reticulum stress, namely adaptation, alarm, and apoptosis (170). Moreover, CCN1 was shown to attenuate TGF-β signaling by scavenging TGF-β, thereby mitigating the overall fibrogenic response *in vitro* and *in vivo* during phases of liver insult (171). In portal myofibroblasts, CCN1 induced ROS formation, p38 phosphorylation, and upregulation of Fas, suggesting that resulting apoptosis requires excessive formation of free radicals and modulation of associated downstream cellular signaling pathways (171). Similarly, the overexpression of CCN2, CCN3, or CCN4 effectively induced ER stress and UPR in HSC, hepatocytes, and portal myofibroblasts suggesting that CCN proteins are generally associated with processes involved in hepatic tissue repair following liver injury (172, 173). Although UPR-mediated hepatocyte apoptosis might hinder hepatic tissue repair, it was suggested that CCN expression might be therapeutically attractive to mitigate liver fibrosis, especially when gene expression is specifically directed to HSC, MFB and portal myofibroblasts (172, 173). Hepatocytes express large quantities of CCN2. This alone does not cause hepatic injury or fibrosis *per se*, but renders the liver more susceptible to injurious actions of other fibrotic stimuli including TGF-β (174, 175). In addition, the reverse (i.e., the blockade of CCN2) has the potential to be an effective treatment for liver fibrosis. This again highlights the complexity in CCN protein biology and demonstrates that targeting of a specific member of this family might result in an unexpected outcome. Therefore, it was proposed that CCN proteins are part of an interconnected team that forms a scaffolding system in which the different CCN proteins act as connectors to permit a balanced series of biological effects (165).

### Angiotensin Stimulated Hepatic Fibrogenesis and Portal Hypertension: Receptor Regulation and Intracellular Signaling

#### Liver Fibrosis and Portal Hypertension

Chronic liver injury drives hepatic fibrosis defined as excessive production and deposition of extracellular matrix (ECM) components (176). In the pathogenesis of liver fibrosis there are two decisive cellular processes, namely recruitment of inflammatory cells leading to perpetuating of inflammation and proliferation of myofibroblasts. They derive mainly from activated HSCs representing the main ECM-producer (177). Inflammation and fibrosis cause narrowing of intrahepatic microvessels and increase intrahepatic resistance to portal blood flow. The intrahepatic hyper-responsiveness of myofibroblasts to vasoconstrictors such as angiotensin II further augments hepatic resistance to portal flow. Thus, progression of fibrosis leads to end-stage liver disease (cirrhosis) and increased portal pressure, both of which are responsible for morbidity and mortality in chronic liver diseases (178). The renin-angiotensin-system (RAS) is crucially involved in the pathogenesis of fibrosis and portal hypertension.

#### Role of Renin-Angiotensin-System in Hepatic Fibrosis and Portal Hypertension

**Figure 11:**
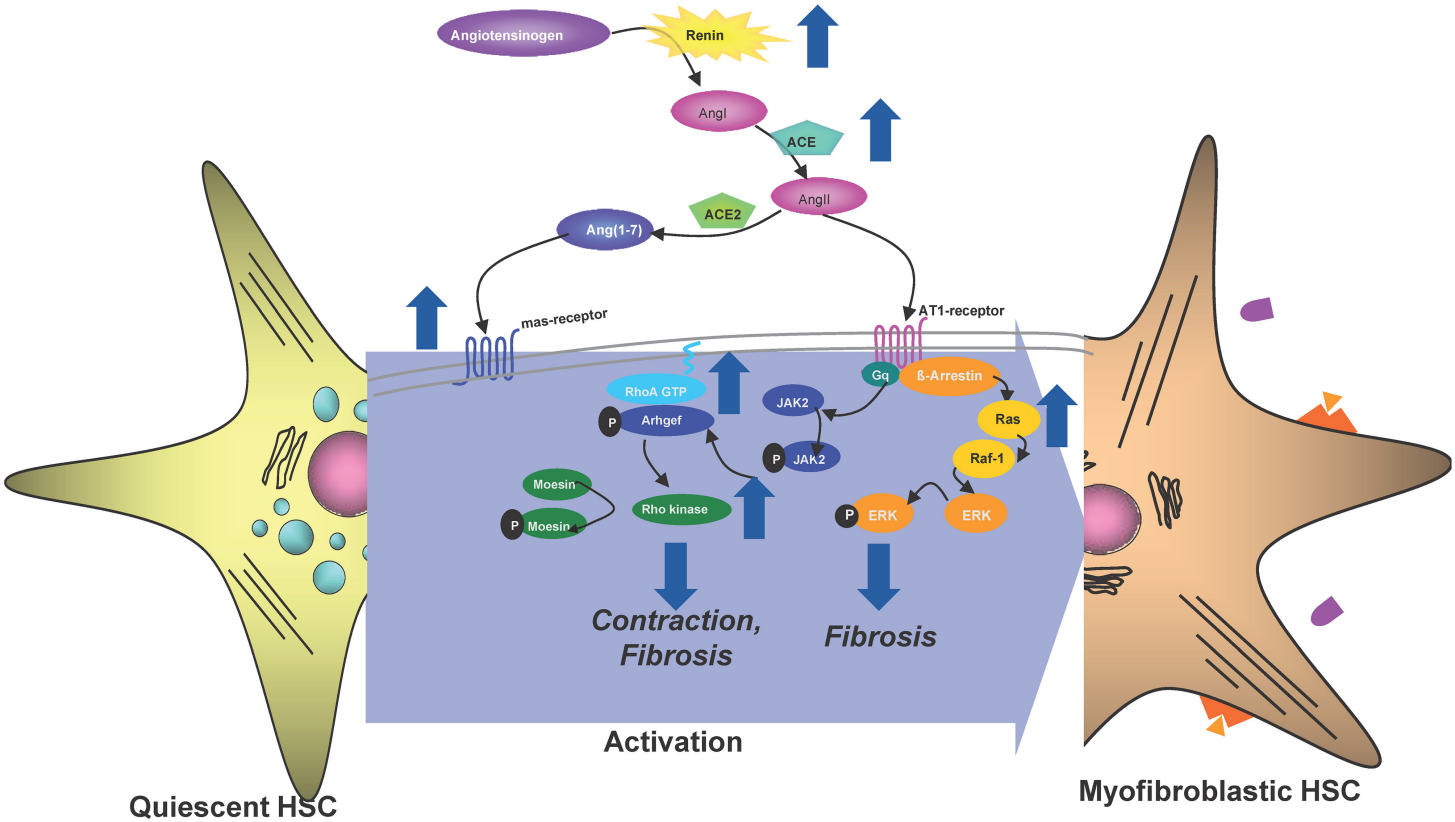
Renin-angiotensin-system (RAS) in liver fibrosis. Angiotensinogen is cleaved by renin into Angiotensin I (Ang I), which is further converted to Angiotensin II (Ang II) by Angiontensin-Coverting-Enzyme (ACE). Ang II is the agonist of AT1R, which signals G-protein dependent *via* Janus-kinase 2 (JAK2), Argef1/RhoA/Rho-kinase. This constitutes the classical RAS, which is known to lead to activation of hepatic stellate cells (HSC) and thereby to fibrosis and their contraction. The G-protein coupled pathway is terminated by beta-arrestin-2 binding to AT1R, which may terminate the contraction, but still may induce fibrosis via ERK-activation. Ang II may be further metabolized to Ang1-7 by ACE2, which represents the alternative RAS-pathway. The alternative RAS-pathway may block contraction via mas-receptor (masR) stimulation. The role of masR and beta-arrestin-2 are still under investigation and be crucial to elucidate the mechanisms in HSC, but also may offer therapeutic options for liver fibrosis and portal hypertension.

In patients and animals with liver cirrhosis, RAS is activated (Figure 11), leading to increased levels of circulating angiotensin II (179). In human liver samples of cirrhotic patients, the components of the classical renin-angiotensin-system (angiotensinogen, renin, angiotensin-converting-enzyme) are upregulated, and Angiotensin-II-type-1 receptor (AT1R) stimulation is increased (180, 181). The link between AT1R stimulation and development of fibrosis with portal hypertension has been well-established in animal models. The continuous injection of angiotensin II induced fibrosis in rats as shown by elevated hepatic hydroxyproline-content. The absence of AT1R in mice resulted in reduced fibrosis upon liver injury, which is in agreement with previous data and supports our working hypothesis that the AT1-receptor is important for the development of fibrosis and portal hypertension. This was underlined in TG(mREN2)27 rats, which overexpress Renin, especially in the liver, and develop spontaneous liver fibrosis and portal hypertension (182) without additional experimental hepatic injury.

On the other side, the alternative RAS, including angiotensin-converting-enzyme-2 and mas-Receptor (masR), is also increased in cirrhotic livers (179, 183). This mediates vasodilation by formation of NO. Thus, the stimulation of the masR using angiotensin (1–7) and/or the non-peptidic orally active agonist AVE 0991 could decrease hepatic resistance and portal pressure in cirrhotic rats (183, 184). Since both receptors are upregulated in liver fibrosis with portal hypertension, it should be important to distinguish interaction of pathways induced by stimulation of the respective receptors (Figure 11).

#### Intracellular Effectors of AT1R in Hepatic Fibrosis

AT1R is coupled to heterotrimeric G-proteins (Gaq/11, Ga12/13) allowing stimulation and activation of several signal pathways (phospholipase C, RhoA, protein kinase C, MAP kinases), which are involved in both smooth muscle contraction, as well as ECM-production. Especially G-protein coupled RhoA/Rho-kinase stimulation of the AT1R-pathway seems to be responsible for fibrosis and portal hypertension (185–187). AT1R stimulation causes G-protein mediated activation of the small monomeric GTPase, RhoA, via GTP-loading and membrane translocation of the protein. GTP-RhoA in turn activates its effector Rho-kinase, which mediates vasoconstriction *via* inhibition of myosin light chain phosphatase. Inhibition of RhoA-activation using statins decreased intrahepatic resistance in cirrhosis and reduced fibrosis as shown in different fibrosis models *in vivo* (185–187). This effect was achieved by inducing p21 dependent senescence in activated hepatic stellate cells/myofibroblast (185–187). Interestingly, the link between AT1R and the RhoA/Rho-kinase pathway is obtained *via* Janus-kinase-2 (JAK2) and Arhgef1, the nucleotide-exchange-factor for RhoA involved in RhoA-activation (180, 181). This could be demonstrated not only in several animal models of liver fibrosis with portal hypertension, but also in liver samples of cirrhotic patients (180–182). As expected, pharmacological inhibition and genetic deletion of JAK2 decreased fibrosis and portal hypertension via downregulation and inhibition of downstream effectors (Arhgef1/RhoA/Rho-kinase) (180, 181) as reported by different groups (188). The G-protein coupled AT1R-pathway is terminated by intracellular binding of beta-arrestin-2, which is also over-expressed in liver fibrosis, probably in HSC, as shown recently (189). The exact role of beta-arrestin-2 in liver fibrosis, beyond a countering AT1R-stimulation, is still under investigation.

masR seems to play a greater role in the regulation of vascular tone than in fibrogenesis, although it is highly upregulated in hepatic fibrosis and likely co-localized with α-SMA, suggesting an expression in activated hepatic stellate cells (179, 183, 184). Intracellular pathways in hepatic stellate cells induced by the masR are still under investigation.

#### HSC-Specific Targeting of AT1R-Downstream Effectors in Portal Hypertension

This increased portal pressure is not only a consequence of increased hepatic resistance in hepatic fibrosis, but is also maintained by splanchnic hyperperfusion as a consequence of decreased splanchnic vascular resistance. This is due to a dysfunctional Rho-kinase pathway among others (190). Therefore, targeting the Rho-kinase system does not only decrease intrahepatic resistance, but also diminishes systemic vascular resistance leading to severe and dangerous hypotension. This may be bypassed by cell-specific targeting of Rho-kinase-inhibitor (Y27632), using different drug-carriers, selectively binding to either M6P/insulin-like growth factor II (M6P/IGFII) receptor or PDGF-R on activated HSC. This strategy decreased portal pressure in different rat models of fibrosis with portal hypertension without major extrahepatic effects (191, 192).

In summary, the role of RAS is complex in hepatic fibrosis and portal hypertension. RAS offers several targets which may be useful in translational approach to treat fibrosis and portal hypertension with distinct effects within and outside the liver.

### Imaging Liver Fibrosis

Diagnosis and staging of liver fibrosis can be done using histopathological stainings of tissue biopsies, using tissue biopsies, circulating biomarkers, multimodal risk scores and non-invasive imaging. Biopsies have remained to be the gold standard, in spite of the fact that they have several drawbacks. These include their invasive nature and the fact that they provide limited spatial information, which leads to sampling variability and which thereby negatively affects diagnostic accuracy (193). Regarding liquid biopsies, several blood biomarker tests are available, including the FibroTest (which assesses the serum levels of α_2_-macroglobulin, apolipoprotein A1, haptoglobin, γ-glutamyl transpeptidase, total bilirubin and alanine transaminase), the ELF test (i.e., the Enhanced Liver Fibrosis test; which assess the serum levels of hyaluronic acid, tissue inhibitor of metalloproteinase-1 and procollagen 3 aminoterminal peptide) and the APRI test (i.e., the aspartate transaminase to platelet ratio index). Imaging biomarkers have thus far mostly relied on the assessment of tissue stiffness using elastography. Ultrasound-based FibroScan analyses have been shown to be reasonably useful for detecting liver cirrhosis, but their accuracy for diagnosing and staging of intermediate to late stage liver fibrosis has its limits (194). The same holds true for magnetic resonance (MR) elastography, as well as for other MRI-based techniques that have been employed for fibrosis diagnosis and staging, such as MR spectroscopy and diffusion-weighted imaging (195). Thus, it would be valuable to possess readily repeatable diagnostic tests for monitoring liver fibrosis progression and treatment response.

**Figure 12:**
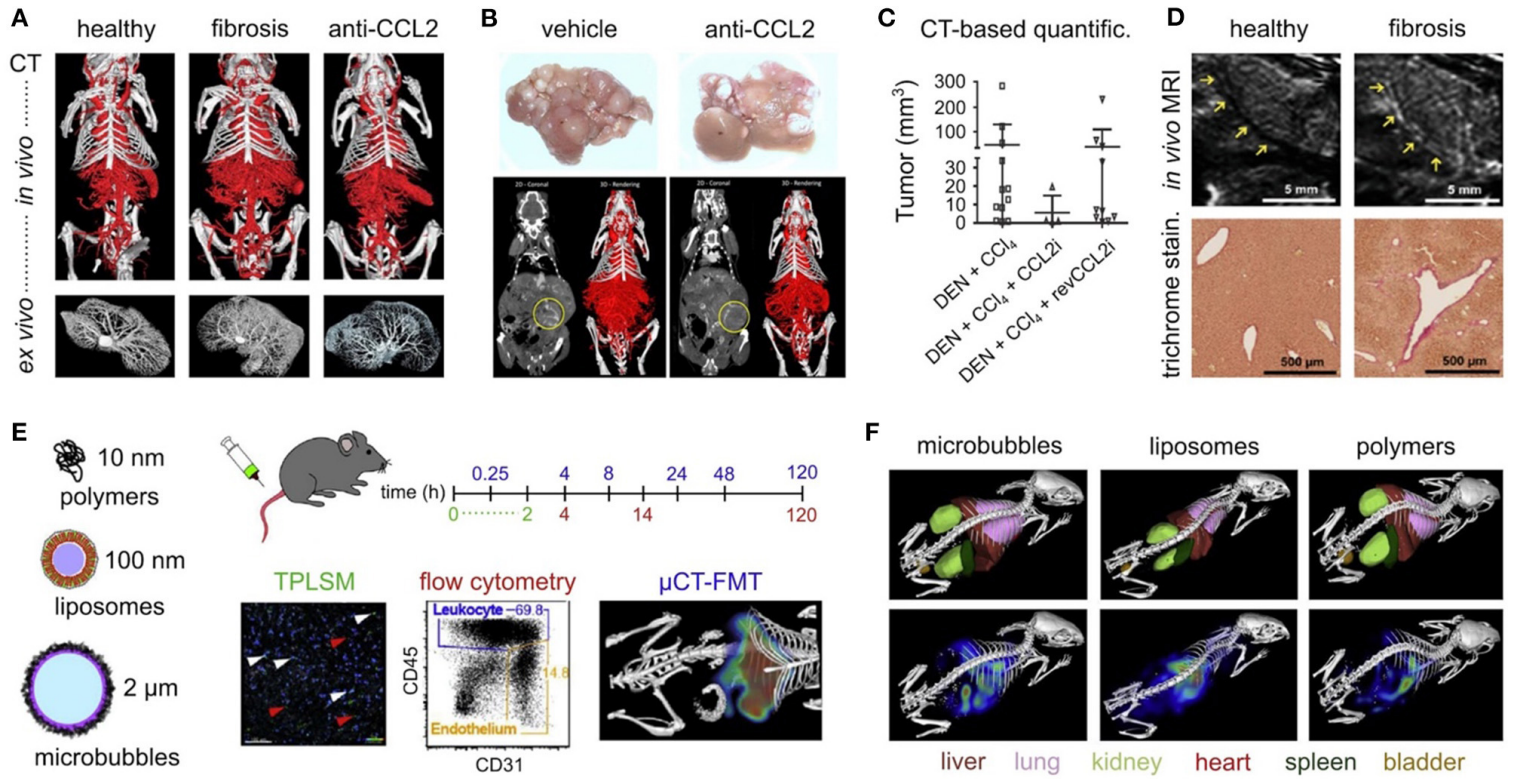
Imaging liver fibrosis. **(A)** Contrast-enhanced *in vivo* and *ex vivo* micro-CT imaging reveals pathological angiogenesis in CCl_4_-induced liver fibrosis, as well as inhibition of fibrosis-associated angiogenesis upon anti-CCL2 RNA aptamer therapy. **(B,C)** Micro-CT-based assessment of the antitumor and anti-angiogenic effect of anti-CCL2 RNA aptamer therapy in the DEN-CCl_4_ fibrosis-HCC mouse model. **(D)** ESMA-enhanced molecular MRI of perivascular elastin deposition in CCl_4_-induced liver fibrosis in mice. **(E,F)** Multimodal optical imaging was employed to demonstrate that CCl_4_-induced liver fibrosis affects the organ distribution and cellular accumulation of prototypic drug delivery systems in the liver. Images reproduced, with permission, from (118, 120, 196, 198, 199).

Contrast-enhanced computed tomography (CT) was employed to study pathological angiogenesis during liver fibrosis progression in mice (196). Both CCl_4_ and BDL mouse models were employed. The methodology for *in vivo* functional and *ex vivo* anatomical blood vessel imaging was adapted from studies in mouse tumor models (197). As shown in Figure 12A, contrast-enhanced CT revealed a correlation between liver fibrosis stage and the hepatic relative blood volume. This was partially explained by the enhanced infiltration of inflammatory monocyte-derived macrophages, which co-localized with newly formed blood vessels. These cells had a pro-angiogenic phenotype, expressing VEGF and MMP9. To attenuate the accumulation of inflammatory macrophages in the liver, an RNA aptamer was employed that binds to CCL2 and inhibits the infiltration of CCR2-positive macrophages. Using both *in vivo* and *ex vivo* CT imaging (Figure 12A), it was demonstrated that blocking inflammatory macrophage infiltration reduced pathological angiogenesis during liver fibrosis progression. It did not, however, affect the overall extent of liver fibrosis. In the combined fibrosis-HCC mouse model (which relies on the application of DEN and CCl_4_), subsequently the contribution of CCR2-positive macrophages to hepatocarcinogenesis and tumor angiogenesis was studied (198). Using transcriptional profiling, three major myeloid cell populations were identified in liver tumors, of which the CCR2-positive subset displayed potent activation of inflammatory and angiogenic signaling pathways. As evidenced by anatomical and functional CT imaging, as well as extensive histology, inhibiting CCR2-positive macrophage infiltration using the CCL2-binding RNA aptamer reduced pathological angiogenesis, hepatic blood volume and liver tumor volume (Figures 12B,C).

Regarding molecular imaging, probes and protocols have been established to address the gradual deposition of ECM components in the liver during fibrosis progression. Caravan et al. combined MRI and the collagen-binding agent EP-3533 for diagnosis and staging of CCl_4_-induced liver fibrosis in mice (200). We employed the elastin-binding agent ESMA, which had been shown to be suitable for imaging atherosclerotic plaque burden by Makowski et al. (201), to non-invasively assess elastin deposition in fibrotic livers. As shown in Figure 12D, it was found that the elastin-binding probe accumulated in perivascular areas in large and medium-sized vessels in fibrotic livers, but not in healthy livers (199). Such targeted molecular imaging setups are considered to be useful for non-invasive and disease-specific diagnosis and staging. Because they are more easily repeatable than biopsies, they can serve as surrogate endpoints in clinical trials, facilitating the development and testing of novel anti-fibrotic drugs.

Drug delivery systems, also known as nanomedicine formulations, are extensively used to improve the biodistribution and target site accumulation of pharmacologically active agents. In the past couple of years, several nanomedicine formulations have been developed for drug targeting in liver inflammation and fibrosis. These have e.g., included 10 nm-sized glycopolymers modified with selectin-ligands (202, 203), 100 nm-sized liposomes loaded with the potent corticosteroid dexamethasone (204, 205), and 2 μm-sized polymeric microbubbles which can be drug-loaded and locally triggered to release their contents using ultrasound (119). By means of hybrid computed tomography—fluorescence molecular tomography (CT-FMT), as well as by fluorescence microscopy and flow cytometry, the biodistribution and the accumulation of these delivery systems in myeloid and lymphoid immune cells was visualized and quantified (Figures 12E,F). This was done in healthy mice a well as in mice with liver fibrosis. Overall, strong uptake in myeloid cell populations was observed, and accumulation in lymphoid cells was minimal (120). Interestingly, while whole-body imaging indicated strong and preferential uptake of all three systems in the liver, flow cytometry and microscopy revealed that macrophage uptake in the liver was significantly reduced in the case of fibrosis (118). Importantly, however, the nanomedicine formulations did still localize in immune cells infiltrates in fibrotic livers, corroborating their propensity to target myeloid cells in areas of inflammation. Last but not least, again using CT-FMT imaging in combination with flow cytometry, it was shown that siRNA-containing nanoformulations efficiently target hepatic stellate cells in fibrotic livers, thereby enabling Cyclin E1-directed gene silencing therapy to attenuate liver inflammation and fibrosis (14).

Taken together, the above examples demonstrate that there has been good progress in establishing materials and methods for liver fibrosis imaging and targeted therapy. In the future, several of these technologies will be evaluated in the clinic, including also theranostic agents, which allow for initial image-based patient stratification and subsequent treatment of pre-selected patient cohorts. Such theranostic probes and protocols can help to make clinical translation more efficient and they are consequently considered to be valuable for successful drug development.

## Concluding Remarks

Hepatic fibrosis is a progressive disease in which the extracellular matrix is accumulating. Cell- and animal-based investigations as well as clinical studies have shown that the progression of hepatic fibrosis is a complex process involving parenchymal and non-parenchymal liver cells, as well as infiltration of immune cells. On a molecular level, the fibrogenic response is driven by numerous soluble mediators that bind to their cognate cell surface receptors and initiate downstream signaling pathways triggering the production and deposition of excessive extracellular matrix compounds. However, despite the important progress in fibrosis research, there is currently no approved anti-fibrotic therapy available that has been ultimately shown to be efficacious in the clinic. The SFB/TRR57 “Organ fibrosis—From Mechanisms of Injury to Modulation of Disease” has made notable achievements in the identification of novel risks factors that aggravate hepatic fibrosis and in the understanding of immunological mechanisms that drive initiation, progression and regression of hepatic fibrosis. Further the research consortium provided novel diagnostic and therapeutic concepts for future clinical management of patients suffering from hepatic fibrosis. In addition, the participating scientists and clinicians of the SFB/TRR57 consortium have bridged the gap between basic science and clinical practice and initiated first clinical trials in which findings of basic science are translated to human pathogenesis and potential clinical applications.

Future will tell, whether the coordinated and collaborative “bench-to-bedside” approach of the SFB/TRR57 helped to establish novel clinically anti-fibrotic therapies.

## Author Contributions

All authors have made a substantial, direct, intellectual contribution to the work by compiling, writing, and editing at least one chapter of this article. All authors read, edited, approved the final version of this paper, and contributed equally to the content of this publication.

## Funding

This article was funded by the German Research Foundation (DFG), SFB/TRR57.

## Conflict of Interest

The authors declare that the research was conducted in the absence of any commercial or financial relationships that could be construed as a potential conflict of interest.

## Publisher's Note

All claims expressed in this article are solely those of the authors and do not necessarily represent those of their affiliated organizations, or those of the publisher, the editors and the reviewers. Any product that may be evaluated in this article, or claim that may be made by its manufacturer, is not guaranteed or endorsed by the publisher.
